# Plant-Derived Nutraceuticals in Mental Health and Brain Function: Mechanisms of Action and Therapeutic Potential

**DOI:** 10.3390/ijms26188849

**Published:** 2025-09-11

**Authors:** Alejandro Borrego-Ruiz, Juan J. Borrego

**Affiliations:** 1 Departamento de Psicología Social y de las Organizaciones, Universidad Nacional de Educación a Distancia (UNED), 28040 Madrid, Spain; a.borrego@psi.uned.es; 2 Departamento de Microbiología, Universidad de Málaga, 29071 Málaga, Spain

**Keywords:** nutraceuticals, phytochemicals, psychiatric disorders, neurodegenerative diseases, functional foods, dietary supplements, nutritional psychiatry, therapeutic tools

## Abstract

Considering the multiple benefits of nutraceuticals, and given the growing interest in exploring these effects, understanding their mechanisms and implications in mental well-being and neurological integrity is essential and requires further examination to clarify their therapeutic potential. This narrative review provides a comprehensive overview of recent advances in plant-derived nutraceuticals, particularly regarding their impact on mental health and brain function, by examining their bioactive components, their involvement in neuropsychiatric conditions, their role in neurodegeneration, emerging nutraceuticals with clinical relevance, and gut microbiome interactions with nutraceuticals and phytochemicals. Essential fatty acids, prebiotics, phytochemicals, and nutrients such as amino acids, vitamins, minerals, and omega-3 fatty acids contribute to mood regulation and cognitive function. Nutraceuticals can prevent or slow neurodegeneration by targeting misfolded proteins and modulating oxidative stress, neuroinflammation, mitochondrial dysfunction, and dysregulated signaling pathways. Phytochemicals act as phytopsychobiotics, influencing mental health through gut microbiome modulation and generation of bioactive metabolites. *Hypericum* and curcumin exert neuroprotective, anti-inflammatory, antioxidant, and antidepressant effects. Ginsenosides promote neuroprotection, partially via gut microbiome-mediated mechanisms. Administration of *Ginkgo biloba* polysaccharides and lavender essential oil improves neurotransmitter regulation, intestinal barrier integrity, and depressive-like behaviors in preclinical models. Omega-3 polyunsaturated fatty acids, anthocyanins, quercetin, catechins, and chlorogenic acid support neuroprotection and cognitive function via modulation of beneficial gut bacteria, short-chain fatty acid production, anti-inflammatory effects, and serotonin metabolism. The landscape of nutraceuticals offers a diverse range of dietary options with considerable potential to promote mental health and prevent neurodegeneration, but further research is required to elucidate how the gut microbiome may enhance these bioactivities.

## 1. Introduction

Nutritional psychiatry is a field that investigates and applies nutritional strategies to promote mental health, focusing on the beneficial effects of diet and nutraceutical functional foods [1]. Mental disorders are characterized by psychological patterns that cause clinically significant distress or impairment, stemming from underlying psychobiological dysfunctions rather than representing usual reactions to everyday stressors or sociocultural influences [1]. Mental disorders are a major contributor to the global health-related burden, with depressive and anxiety disorders consistently ranking among the most disabling conditions worldwide [2]. These disorders affect individuals across all ages, sexes, and regions, posing a significant challenge for public health systems, particularly as recent global events, such as the COVID-19 pandemic, have exacerbated many underlying determinants of poor mental health [2]. In a related manner, neurodegenerative diseases contribute significantly to mental health burden and public health concerns, leading to increasing efforts toward measures aimed at enhancing cognitive performance, particularly among older adults. However, interventions aimed at addressing this specific issue often face limitations in terms of efficacy and applicability. These include methodological heterogeneity, unclear temporal frameworks, limited sample representations, inconsistent definitions of dietary patterns, and cultural variability affecting generalizability [3], highlighting the need for novel or complementary strategies to enhance brain function. Given these challenges, diet-based interventions have emerged as valuable supportive approaches that should be considered as part of comprehensive treatment plans [1].

The term nutraceuticals, coined in the late 1980s by combining nutrition and pharmaceuticals, refers to non-toxic food-derived supplements scientifically demonstrated to provide health benefits for disease prevention and treatment [4]. Over time, the definition of nutraceuticals has expanded to include amino acids, herbs, minerals, phytochemicals, vitamins, and other dietary substances used as supplements in human nutrition [5]. To clarify key distinctions among specific terms such as nutraceuticals, functional foods, dietary or nutritional supplements, medicinal foods, and pharmaceuticals, several authors have highlighted important considerations regarding these related categories [6,7,8,9,10], which can be summarized as follows: (i) functional foods are edible products enhanced with bioactive compounds through breeding, environmental factors, genomic engineering, or fortification, providing health benefits beyond basic nutrition; (ii) dietary or nutritional supplements are concentrated manufactured products, commonly formulated as tablets, powders, liquids, capsules, or soft gels, intended to supplement the diet, and do not necessarily originate from traditional food sources; (iii) medicinal foods require medical supervision and serve as precise dietary management for individuals with specific diseases or conditions, characterized by scientifically established nutritional needs; and (iv) pharmaceuticals derive from the Greek words *pharama* (to charm or enchant) and *phármakon* (magic, cure, potion), referring to products developed through modification of animal or plant sources.

Nutraceuticals provide numerous health benefits due to their content of both essential nutrients, such as carbohydrates, fatty acids, minerals, proteins, and vitamins, and nonessential bioactive food components, including anthocyanins, carotenoids, flavonoids, folates, phenols, polyamines, and non-flavonoid condensed tannins (ellagitannins). These constituents influence key cellular processes, including antioxidant activity, gene expression, cell proliferation, mitochondrial integrity, and immune function enhancement [11,12,13]. Moreover, nutraceuticals have demonstrated protective effects against a wide range of health conditions, such as cancer, obesity, hypertension, cardiovascular disease, gastrointestinal disorders, type 2 diabetes, inflammation, microbial infections, psychotic disorders, spasticity, and ulcers [7,11,12,14,15,16,17]. In this context, the field of nutritional psychiatry has increasingly influenced clinical practice, given the widespread consumption of dietary supplements and nutraceuticals among individuals with and without mental disorders [1].

The complex relationship between nutrition, gut health, and brain function has been extensively acknowledged. The gut–brain axis (GBA) represents a bidirectional communication network that plays a pivotal role in regulating host metabolism, energy balance, immune function, and eating behavior [18]. Disruptions of the GBA, which are often triggered by factors such as inadequate diet, stress, or disease, have been associated with a broad spectrum of health conditions, including malnutrition, mental disorders, and metabolic diseases [19]. Nutraceuticals have emerged as a promising strategy to restore GBA homeostasis by modulating gut microbiome (GM) composition and its associated neuroactive metabolites [20].

Considering the multiple benefits of nutraceuticals, and given the growing interest in exploring these effects, understanding their mechanisms and implications in mental well-being and neurological integrity is essential and requires further examination in order to clarify their therapeutic potential. Based on the principles of nutritional psychiatry, integrating such diet-related tools into clinical practice could be pivotal in promoting overall functionality and mitigating the impact of mental and neurodegenerative disorders. Following these premises and adopting a framework restricted to plant-derived functional compounds, the present narrative review provides a comprehensive overview of recent advances in nutraceuticals, particularly regarding their impact on mental health and brain function, by examining their bioactive components, their involvement in neuropsychiatric conditions, their role in neurodegeneration, emerging nutraceuticals with clinical relevance, and GM interactions with nutraceuticals and phytochemicals.

## 2. Bioactive Components of Nutraceuticals and Functional Foods

The functional food classification encompasses nutraceutical sources such as coffee, fruits, dairy products, soy, tea, and vegetables, as well as fermented foods including barley, kefir, kimchi, kombucha, rice bran, sauerkraut, soy-based products, tempeh, and yogurt. Recommended ingredients for formulation include (i) herbs and spices, such as cayenne pepper, cinnamon, fenugreek, garlic, ginger, red pepper, and turmeric; (ii) honey and its derivatives; (iii) legumes, such as beans, chickpeas, lentils, lupines, navy beans, peanuts, peas, and soybeans; (iv) nuts, such as almonds, Brazil nuts, cashews, hazelnuts, macadamia nuts, pecans, pine nuts, pistachios, and walnuts; (v) seeds, such as those derived from avocado, black cumin, chia, flax, hemp, pumpkin, sesame, and sunflower; (vi) vegetables, such as broccoli, carrots, cauliflower, kale, pumpkin, scent leaves, spinach, tea leaves, and zucchini; and (vii) whole grains, such as amaranth, barley, buckwheat, canary grass, corn, Job’s tears, millet, oats, quinoa, rice, rye, sorghum, Tartary buckwheat, teff, triticale, and wheat. Table 1 summarizes the main categories of functional foods from a nutraceutical perspective, highlighting their principal bioactive components and associated health effects [21,22,23,24,25,26,27,28,29,30,31,32,33,34,35,36,37,38,39,40,41,42,43,44,45,46,47,48,49,50,51,52].

Bioactive constituents, commonly found in foods and agricultural products, are primarily responsible for their nutraceutical functions and associated health benefits. These bioactive compounds contribute to the prevention and management of various chronic diseases by modulating physiological and biochemical processes in the organism [6]. In addition to these established effects, their consistent inclusion in the diet may play a broader role in supporting overall well-being, maintaining long-term health, and potentially mitigating age-related physiological decline, which underscores the importance of considering bioactive-rich foods in daily nutritional strategies [11,12]. Table 2 summarizes the major bioactive components of nutraceuticals, which encompass a diverse range of substances, including carotenoids, dietary fiber and prebiotics, essential fatty acids, essential minerals, and phytochemicals such as polyphenols, probiotics, and vitamins.

Minerals play a pivotal role in maintaining human homeostasis and metabolic function. Mineral deficiencies have been implicated in numerous pathological conditions, making adequate mineral intake essential for optimal health [53]. Essential minerals such as calcium, chloride, magnesium, phosphorus, potassium, sodium, and sulfur, along with trace minerals including cobalt, copper, fluoride, iodine, iron, manganese, selenium, and zinc, contribute to a variety of nutraceutical functions. Growing evidence supports the significant health benefits of these minerals. For instance, iodine fortification of salt has markedly reduced the prevalence of goiter [54], selenium is integral to selenoproteins that regulate reactive oxygen species (ROS) and maintain redox balance across tissues, while zinc is crucial for immune function and cellular growth, with its deficiency compromising cellular homeostasis and increasing susceptibility to allergies, autoimmune diseases, cancer, and infections [55].

Certain vitamins, notably vitamins E, A, D, and C, are well-established antioxidants. Consumption of foods rich in these vitamins has been shown to improve overall health and serve as a preventive measure against a variety of conditions, including heart disease, vision loss, cancer, osteoporosis, immune system suppression, osteoarthritis, type 2 diabetes, and cardiovascular disease [56]. These antioxidant vitamins mitigate the harmful effects of ROS and free radicals by inhibiting oxidative processes [57].

Essential fatty acids (EFAs) constitute indispensable fatty acids that the human organism is not capable of synthesizing and must be obtained through the diet. The two primary EFAs with significant nutraceutical properties are the omega-3 fatty acid α-linolenic acid and the omega-6 fatty acid linoleic acid, both of which are involved in metabolic pathways linked to inflammation, cellular function, behavior, and mood regulation [58,59]. EFAs contribute to a range of physiological processes, including cardioprotection, immune modulation, antimicrobial activity, metabolic regulation, anti-inflammatory effects, and overall health promotion [60].

Dietary fiber consists of polysaccharides found in various plant-based foods. Soluble fibers include mucilage, β-glucan, and pectin, whereas cellulose, hemicellulose, and lignin are classified as insoluble fibers. Incorporating dietary fiber into food products has been shown to enhance their nutraceutical value, nutritional profile, structural integrity, shelf life, and sensory attributes [61]. When not fully digested or absorbed in the small intestine, dietary fiber primarily functions as a substrate for the fecal microbiota in the large intestine, contributing to the prevention of numerous diseases and health conditions. In addition, fiber supports detoxification processes and helps regulate lipid and glucose levels in the bloodstream, thereby reducing the risk of cardiovascular disease, obesity, and type 2 diabetes [61,62]. Dietary fibers include prebiotics, defined as plant carbohydrates that resist digestion and absorption in the small intestine. These prebiotics promote the production of microbial metabolites by the GM. Common prebiotics encompass resistant starch, oligosaccharides (e.g., galactooligosaccharides, inulin, and fructooligosaccharides), guar gum, and pectin. Acting as substrates for beneficial colonic bacteria, prebiotics improve colonic pH and stimulate the production of short-chain fatty acids (SCFAs). SCFAs provide energy to mucosal cells and enhance mucosal blood flow. Moreover, they exhibit antioxidant properties by scavenging ROS, anti-proliferative effects through regulation of apoptosis, signal transduction, and gene expression, as well as anti-inflammatory effects by reducing prostaglandin E2 and pro-inflammatory cytokines [63,64,65].

A wide variety of foods with nutraceutical properties, such as vegetables, medicinal herbs, fruits, whole grains, and beans, contain significant phytochemicals that have been shown to provide health benefits, including the enhancement of cognitive function [5,66,67,68]. However, some compounds, including polyphenols, alkaloids, glycosides, terpenes, and terpenoids, can inhibit nutrient absorption and are therefore classified as anti-nutrients [69].

Probiotics have been shown to provide significant health benefits and hold a pivotal role in the food industry. They are widely incorporated into various products, including probiotic foods, pharmaceutical formulations, animal feeds, infant formulas, and dietary supplements [70]. Probiotic cultures are commonly found in fermented dairy products, probiotic-enriched foods, and other fermented products, most of which are consumed orally [71]. The mechanisms by which probiotics exert their effects are diverse and include competitive inhibition of pathogenic microorganisms, antimicrobial activity, alleviation of gastrointestinal disorders, immune system modulation, and beneficial impacts on metabolic, neurological, and mental health conditions [72,73,74,75,76,77,78,79].

**Table 2:** Major bioactive components of nutraceuticals.

Bioactive Component	Nutraceutical Effects	References
Minerals	- Minerals fulfill a variety of biological and physiological functions and have considerable potential in the maintenance of homeostasis and metabolism.	[53]
Vitamins	- Vitamins play a significant role in the prevention of various diseases and health conditions, including heart disease, vision loss, cancer, osteoporosis, immune system suppression, osteoarthritis, type 2 diabetes mellitus, and cardiovascular diseases. - Vitamins enhance quality of life and play a role in the oxidation process, impeding the actions of reactive oxygen species and free radicals, implicated in many acute and chronic diseases.	[56,57]
Essential fatty acids	- Essential fatty acids support a multitude of critical functions in humans, including cardioprotective, immune-boosting, antimicrobial, metabolic, cardiovascular-promoting, reduced risk of atherosclerosis, anti-inflammatory, and health-promoting effects. - Adequate intake of essential fatty acids enhances brain health and cognitive function, alleviates depression, and mitigates pain.	[58,60]
Dietary fiber	- Fiber binds with excess fat and glucose, facilitating their release via stool, and also mitigates the risk for cardiovascular diseases, obesity, and type 2 diabetes mellitus. - Fiber plays a crucial role in the body’s detoxification processes and prevents diverticulosis.	[61,62]
Prebiotics	- Prebiotics function as substrates for beneficial bacteria, increasing their growth, enhancing colon-pH reduction, and increasing bacterial short-chain fatty acid production. Short-chain fatty acids possess antioxidant, anti-proliferative, and anti-inflammatory properties.	[63,65]
Phytochemicals	- Many phytochemicals present antioxidant, cardioprotective, immune-boosting, antimicrobial, metabolic, heart-promoting, anti-inflammatory, and health-promoting properties.	[67,68]
Probiotics	- Probiotics are associated with the prevention and/or treatment of a wide range of diseases and conditions, including diarrhea, Crohn’s disease, lactose intolerance, *Helicobacter pylori* infection, ulcerative colitis, metabolic diseases (e.g., dyslipidemia, obesity, and diabetes mellitus), impaired immunity, respiratory tract infections, allergies (e.g., eczema and asthma), neurological and mental health conditions (e.g., depression and anxiety), bacterial vaginosis, high blood pressure, and inflammatory bowel disease.	[71,73,74,75,76,77,78,79,80,81]

## 3. Nutraceuticals in the Context of Mental Health Conditions

The importance of natural bioactive compounds with potential health-promoting effects is increasingly recognized. As a result, diet-based approaches for managing and preventing chronic diseases, including mental disorders, are gaining prominence. In recent decades, growing evidence has shown that diets rich in nutraceuticals, such as fiber, phytochemicals, and omega-3 fatty acids, are linked to improved mental health and a reduced risk of neurodevelopmental disorders [82]. Advances in our understanding of mental disorders and their underlying molecular mechanisms further indicate that deficiencies in key nutrients, including amino acids, vitamins, minerals, fiber, and omega-3 fatty acids, are associated with an increased risk of mental illness [83].

Amino acid supplementation has been recognized as an important therapeutic approach for various mental disorders, including depression. Studies have shown that methionine, phenylalanine, tryptophan, and tyrosine can reduce the risk of depression [84]. Serotonin and its precursor tryptophan are well-established as key regulators of sleep and mood. Clinical evidence indicates that tryptophan supplementation can restore serotonin levels and significantly alleviate depressive symptoms [85]. Tyrosine and phenylalanine, via their conversion to dopamine and norepinephrine, also exhibit notable antidepressant effects [86]. Furthermore, methionine, particularly in combination with ATP, has been reported to exert significant antidepressant effects [87].

Deficiencies in vitamin B12 and folate (vitamin B9) have been reported in individuals with depression and bipolar disorder (BD), and supplementation with these vitamins has been associated with a significant reduction in both depressive and manic episodes [88]. Several clinical trials have also demonstrated that vitamin B6 and folic acid are effective in the management of anxiety, panic disorder, and obsessive–compulsive disorder [89]. In addition, supplementation with vitamins C and E has been shown to slow the progression of Alzheimer’s disease (AD). Specifically, male individuals who regularly consumed these vitamins through dietary supplements exhibited improved cognitive function and overall mental performance [90].

Zinc is an essential trace element involved in numerous biochemical and physiological processes that are pivotal for brain development and function [91]. Evidence links insufficient zinc intake to a higher risk of depression, with studies showing an association between low zinc levels and the onset of depressive symptoms [92,93]. Magnesium plays a crucial role in various brain processes, including maintaining neuronal membrane fluidity, which is essential for stable brain function [94]. Multiple studies have reported an inverse relationship between magnesium intake and depression risk, and serum magnesium levels are consistently lower in patients with major depression than in healthy controls [95,96]. The role of copper in depression has been associated with inflammatory responses, oxidative stress, and synaptic plasticity. However, due to its redox activity, copper can catalyze the formation of ROS, which makes its uptake subject to regulation [97]. Evidence also suggests that patients with major depressive disorder (MDD) exhibit higher serum copper levels than healthy individuals [98]. Iron is essential for normal brain development and function [99], and its deficiency has been linked to an increased risk of depression [100]. Although the underlying mechanisms remain unclear, research suggests that altered iron status may influence brain-derived neurotrophic factor (BDNF) levels and oxidative stress, which in turn affect brain development, neural regeneration, synaptic transmission and plasticity, and neurogenesis [101].

Several minerals have been investigated as therapeutic agents for alleviating depressive symptoms. Selenium, an essential trace element, is critical for numerous physiological processes, including thyroid hormone metabolism, antioxidant defense, and immune regulation [102]. Higher selenium levels have been associated with a lower prevalence of depression, likely through mechanisms such as antioxidant effects, reducing malondialdehyde levels, acetylcholinesterase (AChE) activity, and lipopolysaccharide (LPS)-induced oxidative damage, as well as anti-inflammatory effects, modulating dopamine and norepinephrine secretion, and also suppressing microglial activation [97]. Treatments using selenium have demonstrated that this mineral can activate the JAK2/STAT3 pathway, restore dopamine and norepinephrine levels, reduce IL-1β secretion, and increase viable cortical neuron counts, thereby mitigating fluoride-induced depressive-like behaviors [103].

Lithium, as a psychotropic agent, has been widely employed for the treatment of BD, demonstrating effectiveness in preventing suicidal behavior as well as depressive and manic episodes [104]. The proposed mechanisms underlying the antidepressant effects of lithium include (i) stimulation of hippocampal neurogenesis, thereby enhancing neuroplasticity; (ii) elevation of serum BDNF levels in patients with depression; and (iii) protection of the blood–brain barrier (BBB) and neurovascular unit from structural and functional damage [97].

In addition, fiber deficiency can alter the composition and activity of the GM, leading to changes in SCFA production. SCFAs influence tryptophan availability and vagal nerve activation, which can modulate brain inflammatory signaling and, in turn, affect cognition, behavior, and neuronal coordination [105,106,107]. Numerous studies have reported associations between neuropsychiatric and neurodegenerative disorders, such as BD, MDD, Parkinson’s disease (PD), AD, functional disorders, and autoimmune conditions (e.g., multiple sclerosis), and neuroinflammation. Thus, the onset and progression of these conditions are strongly influenced by GM activity [106,108,109].

### 3.1. Polyunsaturated Omega Fatty Acids

Evidence on the effects of nutraceuticals on psychiatric symptoms and disorders remains overall limited, except in the case of omega-3 fatty acids [110,111]. Omega-3 polyunsaturated fatty acids (PUFAs) are key components of neuronal cell membranes, particularly in dendritic and synaptic regions, where they are incorporated into phospholipids and cholesterol esters. Omega-3 PUFAs influence synaptic membrane phospholipid composition and modulate second messenger cascades [112], affecting both dopaminergic and serotonergic signaling pathways [113]. Specifically, eicosapentaenoic acid (EPA) and docosahexaenoic acid (DHA) play pivotal roles in regulating neurogenesis, promoting cell survival, and facilitating neurotransmission [114,115].

In humans, inadequate dietary intake of omega-3 PUFAs has been linked to an increased risk of several psychiatric disorders, including attention-deficit/hyperactivity disorder (ADHD), autism spectrum disorder (ASD), BD, dementia, depression, and schizophrenia. EPA and DHA, in particular, appear to be essential for maintaining mental health, and their deficiency has been implicated in the pathophysiology of the aforementioned conditions [116]. These effects may involve the modulation of inflammatory processes, as well as direct influences on neuronal membrane fluidity and receptor function [117].

A growing body of research has highlighted the potential benefits of α-linolenic acid, which is found in plant sources such as blackcurrant seed oil, olive oil, and evening primrose oil, for the prevention and treatment of mental health disorders, including depression and schizophrenia, as well as for supporting neurological function [118,119,120]. This acid has also been shown to reduce neuronal apoptosis through multiple mechanisms, including the attenuation of ROS-mediated damage, which exerts anti-apoptotic effects via upregulation of anti-apoptotic proteins coupled with downregulation of pro-apoptotic proteins, thereby decelerating neuronal loss by apoptosis [121].

### 3.2. Hyperforin

Another notable nutraceutical is hyperforin, a bioactive compound extracted from St. John’s wort (*Hypericum perforatum*). Hyperforin inhibits the reuptake of several neurotransmitters, including dopamine, gamma-aminobutyric acid (GABA), L-glutamate, norepinephrine, and serotonin [122]. Its effects are also mediated through activation of transient receptor potential C6 (TRPC6) channels, which promotes Na^+^ influx and triggers noradrenaline release [123,124]. Hyperforin-induced inward Ca^2+^ currents via TRPC6 have been shown to enhance neuroplasticity, including axonal sprouting and increased dendritic spine formation and density [125]. In 28 healthy volunteers, administration of 2% hyperforin facilitated long-term depression-like synaptic plasticity [126].

Evidence indicates that standard extracts of *Hypericum* spp. or hyperforin itself can reduce inflammation and oxidative stress, processes implicated in the progression of certain mental health conditions [127,128]. Bonaterra et al. [129] underscored the protective effects of *H. perforatum* extract against glutamate/N-methyl-D-aspartate (NMDA) or cortisol-induced excitotoxicity, its anti-inflammatory potential in LPS-activated human macrophages, and its ability to promote neurite outgrowth. In addition, the extract also protected hippocampal and microglial cells from activation by pro-inflammatory cytokines, including IL-1β and tumor necrosis factor-α (TNF-α), as well as from H_2_O_2_-induced ROS and oxidative stress. Hyperforin has been shown to inhibit 5-lipoxygenase, thereby reducing the synthesis of pro-inflammatory leukotrienes in human neutrophils [130]. Additional studies have demonstrated the antioxidant and anti-inflammatory properties of hyperforin through inhibition of AChE and butyrylcholinesterase activity [131,132]. Marrelli et al. [127] further confirmed that the neuroprotective, anti-inflammatory, and antioxidant effects of *Hypericum* spp., and their antidepressant-like activity, are not limited to hyperforin alone, as other bioactive compounds in *H. triquetifolium* and *H. neurocalycinum* contribute to these effects [133]. The recent identification of two novel prenylated β-diketone compounds (A and B) in *H. perforatum*, with compound A showing potent AChE inhibition, further reveals the anti-dementia potential of *Hypericum*. Specifically, hyperforin has been shown to reduce β-amyloid production and Tau phosphorylation in PC12 cells, while exerting anti-apoptotic, anti-inflammatory, and antioxidant effects in mouse models of AD [134,135,136].

### 3.3. Curcumin

Curcumin (diferuloylmethane), which constitutes the primary bioactive compound in turmeric (*Curcuma longa*, family *Zingiberaceae*), has been shown to enhance serotonin and dopamine release while inhibiting glutamate release. These mechanisms may underlie its efficacy in mood disorders and Huntington’s disease [137,138,139]. The novel curcumin derivative J147, initially developed for AD, exhibits antidepressant properties and anxiolytic-like effects, likely through enhanced monoamine neurotransmission [140]. As noted by Ramaholimihaso et al. [139], curcumin can modulate multiple inflammatory pathways implicated in mental health conditions and neurodegenerative disorders. Experimental studies indicate that curcumin inhibits IL-1, TNF-α, nuclear factor kappa-light-chain-enhancer of activated B cells (NF-κB), P2X7-mediated NLRP3 inflammasome activation, cyclooxygenase-2 (COX-2), and nitric oxide synthase, which in turn affects indoleamine 2,3-dioxygenase activity. Beyond its anti-inflammatory effects, curcumin exerts neuroprotective effects through activation of the PI3K/AKT/GSK-3 and PI3K/AKT/CREB/BDNF signaling pathways, as well as through the reduction in oxidative stress [139,141].

Clinical studies focusing on the effects of curcumin in depression exhibit considerable heterogeneity in study design, dosing, and formulations aimed at enhancing bioavailability, ultimately resulting in inconsistent findings [139]. In addition, definitive clinical evidence regarding the neuroprotective efficacy of lipidated curcumin in AD remains limited. Nevertheless, preclinical studies have highlighted the neuroprotective potential of curcumin, including the upregulation of heme oxygenase in various cell lines, reduction of soluble Tau oligomers, and improvement of cognitive function through increased expression of heat shock proteins HSP70, HSP90, and HSC70 [142,143]. The curcumin derivative J147 has been proposed as an allosteric modulator of mitochondrial α-F1-ATP synthase, enhancing intracellular calcium levels and activating the AMPK/mTOR pathway, a key regulator of cellular longevity [144]. Another curcumin component, the essential volatile oil curcumol, exhibits poor bioavailability but functions as an allosteric modulator of GABA(A) and glycine receptors, thereby exerting an inhibitory control mechanism distinct from benzodiazepines [145]. Curcumol also inhibits glycine-activated currents [146], which may reduce hippocampal tonic inhibition, a pathway crucial for cognitive performance [122].

A substantial body of research has explored the therapeutic potential of curcumin. Nevertheless, its clinical application is constrained by notable safety and pharmacokinetic challenges that limit both bioavailability and efficacy [147]. The compound exhibits an excellent safety profile, and both the Joint FAO/WHO Expert Committee on Food Additives and the European Food Safety Authority have established an Acceptable Daily Intake of 0–3 mg/kg body weight [148]. Although clinical trials in healthy individuals have generally confirmed its safety, prolonged administration has occasionally been associated with adverse effects such as nausea, diarrhea, headache, rash, and yellow stool [149]. The pharmacokinetic limitations of curcumin are multifactorial. For instance, its poor aqueous solubility significantly restricts gastrointestinal absorption. This specific limitation is compounded by extensive first-pass metabolism, during which curcumin undergoes rapid conjugation via glucuronidation and sulfation [150]. Consequently, the majority of circulating curcumin is pharmacologically inactive, with only a small fraction existing in metabolically active forms [151].

### 3.4. Silexan

Silexan is a medicinal preparation derived from *Lavandula angustifolia* that contains essential oils such as linalool and linalyl acetate. This product has been approved in multiple countries for the treatment of generalized anxiety disorder (GAD), with clinical trials demonstrating considerable efficacy. Consistent with other plant-based therapies, Silexan exhibits multicomponent and multitarget effects, contributing to its intrinsic antidepressant-like properties in patients with comorbid depressive or mixed anxiety–depressive disorders [152]. Beyond shared serotonergic mechanisms with conventional anxiolytics and antidepressants, Silexan has been shown to moderately inhibit T- and N-type calcium channels, producing effects similar to gabapentin but without its associated side effects. Furthermore, Silexan promotes neuroplasticity by activating cAMP response element-binding protein (CREB) via protein kinase A (PKA) and mitogen-activated protein kinase (MAPK) signaling pathways, while also enhancing neurite outgrowth and synaptogenesis, processes considered common final pathways of antidepressant action [152,153]. Notably, in addition to linalool and linalyl acetate, approximately 40 other compounds present in lavender essential oil, including β-caryophyllene and its derivatives, have been shown to reduce the mRNA expression of amyloid precursor protein and tau in Aβ1-42-treated SH-SY5Y neuronal cells [154,155].

### 3.5. Ginkgo biloba Extracts

Among nutraceuticals and bioactive dietary compounds, the standardized *Ginkgo biloba* (Gb) extract EGb 761 is the most extensively studied herbal product in clinical trials for cognitive impairment, dementia, and AD [156]. The use of Gb (family *Ginkgoaceae*) leaves and seeds in traditional Chinese medicine has a long history across centuries, primarily for the treatment of respiratory conditions, tinnitus, and vertigo. Moreover, there is also evidence supporting its efficacy in mitigating age-related cognitive decline and memory deficits [157].

Gb contains a range of bioactive compounds, including terpenoids (ginkgolides A, B, and C), polyphenols, organic acids, and flavonoids such as quercetin, kaempferol, ginkgetin, and isorhamnetin [158]. These constituents have been associated with anti-inflammatory, antioxidant, and neuroprotective effects, making them valuable for addressing cognitive function and alleviating symptoms of AD and vascular dementia [159,160]. The flavonoids in Gb extracts inhibit the activity of metabolic enzymes, resulting in decreased prostaglandin levels and a consequent reduction of pro-coagulant and pro-inflammatory activity. They also exhibit vasodilatory effects and potent scavenging activity against ROS, thereby mitigating oxidative damage in the central nervous system (CNS). The diterpene ginkgolides A, B, and C are characteristic of Gb and act as antagonists of platelet-activating factor, reducing the risk of thrombosis, including cerebral thrombosis, and improving cerebral blood flow [157,161]. Furthermore, Gb extracts have been shown to enhance endothelial nitric oxide production, thereby exerting subsequent effects on both peripheral and cerebral blood circulation [162].

Polyphenols can exhibit either antioxidant or pro-oxidant cytotoxic effects depending on their concentration. At high concentrations, polyphenols have been shown to inhibit Nrf2 signaling pathways and the expression of several key antioxidant enzymes, including glutathione peroxidase, glutathione transferase, NAD(P)H-quinone oxidoreductase, sirtuin-1, and thioredoxin. These enzymes are pivotal for regulating ROS metabolism, detoxifying xenobiotics, and suppressing cancer cell progression through mechanisms such as apoptosis and cell cycle arrest, in accordance with hormesis pathways [163,164].

The diterpenes ginkgolides A, B, and C have been shown to inhibit COX-2 and 5-lipoxygenase (5-LOX), thereby reducing the production of pro-inflammatory mediators and modulating proteins involved in oxidative stress, such as peroxiredoxin 1 [157,158]. Gb also contains phytoconstituents, including bilobalide, isorhamnetin, and quercetin, which have been associated with anti-inflammatory effects and a reduction in ROS production [165]. Quercetin and bilobalide act as free radical scavengers, while bilobalide has been correlated with upregulation of cytochrome c oxidase subunit III and BCL-2 expression, suggesting anti-apoptotic activity [166]. Isorhamnetin has been demonstrated to inhibit DNA fragmentation and apoptosis, further contributing to the neuroprotective properties of Gb [158,165].

EGb 761 has been shown to inhibit the generation of hydrogen peroxide radicals, superoxide anion radicals, ROS, reactive nitrogen species, peroxyl radicals, and hydroxyl radicals [167]. In addition, EGb 761 promotes the upregulation of antioxidant enzymes, including glutathione peroxidase and superoxide dismutase [168,169]. Similarly, Gb extract exhibits a broad pharmacological profile that encompasses the prevention of oxidative stress, thereby protecting the CNS from depressive processes while concomitantly enhancing aspects related to neuroplasticity and mitochondrial function. Furthermore, Gb has been observed to modulate multiple neurotransmitter systems. Specifically, it exerts inhibitory effects on monoamine oxidase A, reduces norepinephrine uptake, enhances 5-hydroxytryptamine uptake in synaptosomes, and increases extracellular dopamine and noradrenaline levels [170,171,172].

### 3.6. Ginseng Extracts

Ginseng (*Panax ginseng*) is possibly one of the most widely consumed herbal nutritional products worldwide. The root of *P. ginseng* has a long history of use in traditional medicine and is incorporated into various culinary preparations, food products, and beverages. In addition to its dietary applications, the root is also utilized in pharmaceutical formulations for its medicinal properties [173]. Traditionally, ginseng is employed to promote rejuvenation and longevity, as well as to counteract frailty, stress, and both mental and physical fatigue [174]. Moreover, it is used as an adjunctive treatment for diverse health conditions, such as diabetes and cardiovascular disease [173,175]. In this respect, several systematic reviews have been conducted to assess the clinical evidence regarding the effectiveness of ginseng across a range of health conditions, including erectile dysfunction [176], chronic fatigue [177], hypertension [178], diabetes [179], and AD [180], and as an ergogenic aid [181].

**Figure 1:**
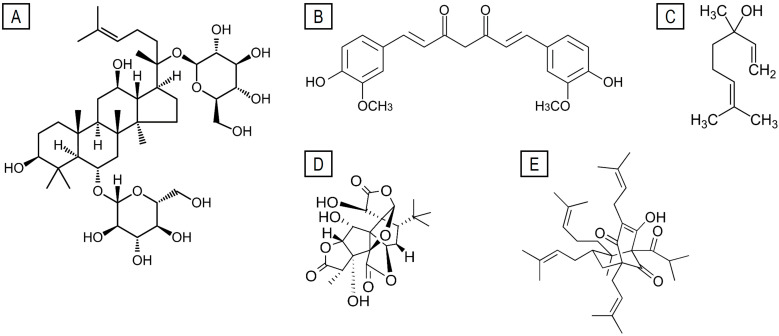
Chemical structure of several phytochemicals. (**A**) Triterpenoid saponins present in ginsenosides, which possess a triterpenoid skeleton and are classified into groups such as protopanaxadiol (PPD), protopanaxatriol (PPT), and others, distinguished by the structure of their aglycone (non-sugar moiety) and the type and position of attached sugar residues. (**B**) Curcuminoids, a class of lipophilic phenolic compounds, feature a linear diarylheptanoid structure with two aromatic rings connected by a seven-carbon chain. (**C**) Linalool, an acyclic monoterpene alcohol derived from plant essential oils, which has the chemical formula C_10_H_18_O, containing two double bonds and a hydroxyl functional group. (**D**) Ginkgolides are diterpenoid cage-like molecules comprising six five-membered rings, three lactone rings, a tetrahydrofuran moiety, and a characteristic tert-butyl group. (**E**) Hyperforin, which constitutes a polycyclic polyprenylated acylphloroglucinol (PPAP) derivative, consists of a bicyclic phloroglucinol core with lipophilic prenyl (isoprene) chains.

A unification of traditional properties with modern medical theory is being achieved through pharmacological and clinical evidence [182]. However, further investigation is required to explore novel indications, particularly in the domain of cognitive enhancement. Ginseng contains a multitude of active constituents, the most significant for cognition being ginsenosides, which are dammarane-type saponins. These can be categorized into two primary subtypes: protopanaxadiol (PPD) and protopanaxatriol (PPT). Ginsenoside Rg1, which is a member of the PPT subtype, interacts with a variety of biological targets and signaling pathways, including peroxisome proliferator-activated receptors (PPARs), MAPK, Toll-like receptors, arachidonic acid metabolism, and apoptosis [175]. With respect to cognition, Rg1 has demonstrated memory-enhancing and neuroprotective effects in models of AD and amyloidogenesis [183]. Studies involving punitive stimuli have shown that Rg1 can reduce memory impairment induced by chronic stress, potentially via regulation of the BDNF/TrkB/ERK pathway in the prefrontal cortex [184]. In addition, Rg1 has been found to ameliorate sleep deprivation-induced memory deficits in murine models, likely by mitigating oxidative stress in the cortex and hippocampus [185]. On the other hand, ginsenoside Rb1, which is derived from the PPD subtype, enhances spatial memory and mitigates memory impairment in animal models [183]. It also ameliorates cognitive dysfunction induced by anesthetics and surgical procedures through suppression of neuroinflammation and oxidative stress [186]. Other PPT ginsenosides, including Rh1 and Rh2, possess anti-apoptotic, anti-angiogenic, and antioxidant properties, which have direct implications for memory and learning. Specifically, Rh1 can mitigate sleep deprivation-induced cognitive impairments [185,187], while Rh2 attenuates neuroinflammation and oxidative stress, key contributors to neurodegeneration [188]. These findings underscore the therapeutic potential of Rh1 and Rh2 in counteracting neurodegenerative processes. Figure 1 presents a schematic representation of the chemical structures of several phytochemicals, including triterpenoid saponins found in ginsenosides, essential oils such as curcuminoids, linalool, ginkgolides, and phenolic compounds such as hyperforin.

## 4. The Role of Nutraceuticals in Neurodegeneration

Neurodegenerative disorders predominantly arise from the abnormal misfolding of tau and amyloid-β (Aβ) proteins, a process that is central to the progression of AD. Traumatic brain injury has been associated with alterations in tau, trans-active response DNA-binding protein-43 (TDP-43), and Aβ. Dysfunction of tau and TDP-43 can also contribute to epilepsy and other tauopathies. In Down syndrome, Aβ is a major driver of a cytotoxic cascade of molecular and cellular events, whereas in PD, α-synuclein plays a comparable role, ultimately resulting in further degeneration [189].

Misfolded proteins have been shown to promote NF-κB activation, which in turn stimulates the production of pro-inflammatory cytokines, including TNF-α and IL-1β. This inflammatory response triggers the expression of harmful mediators such as COX-2 and inducible nitric oxide synthase (iNOS). These effects are driven by the release of ROS and glutamate-induced oxidative stress, ultimately leading to mitochondrial dysfunction and cellular toxicity [190]. In addition, misfolded proteins disrupt glycogen synthase kinase-3β (GSK3β) signaling, promoting the production of inflammatory cytokines. This dysregulation contributes to tau hyperphosphorylation and increased cholesterol synthesis, resulting in the formation of lipid rafts, which facilitate abnormal protein processing and misfolding by enhancing specific enzymatic activities, thereby establishing a self-perpetuating cycle. Furthermore, protein misfolding interferes with multiple signaling pathways, including extracellular signal-regulated kinase (ERK), cAMP response element-binding protein (CREB), and protein kinase A/protein kinase B (PKA/PKB), as well as cholinergic neurotransmission. These alterations have been linked to impaired cognitive performance and synaptic degeneration [189].

**Figure 2:**
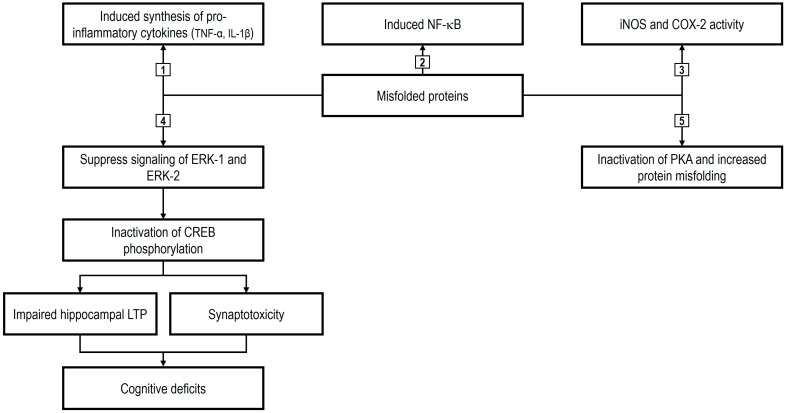
Summarized pathogenesis of misfolded proteins and neurodegeneration mediated upon their activation. NF-κB: nuclear factor kappa-light-chain-enhancer of activated B cells; iNOS: inducible nitric oxide synthase; COX: cyclooxygenase (COX); PKA: protein kinase A; ERK: extracellular signal-regulated kinase; CREB: cyclic adenosine monophosphate (cAMP) response-element binding signaling; TNF-α: tumor necrosis factor-α; LTP: hippocampal long-term potentiation. The squares that contain numbers indicate the targets of nutraceuticals through the following mechanisms: (1) prevention of pro-inflammatory cytokine activation; (2) inhibition of NF-κB activation; (3) inhibition of COX and iNOS pathways; (4) prevention of ERK-1 and ERK-2 signaling; and (5) modulation of PKA/PKB pathways. Inhibition of these cascade proteins by active nutraceuticals tends to provide neuroprotection.

Nutraceuticals have been shown to modulate cellular and molecular cascades, thereby preventing neurodegeneration by targeting misfolded proteins at multiple levels and serving as adjunctive therapeutic agents. Research has demonstrated that these compounds possess antioxidant, cholesterol-lowering, and anti-inflammatory properties and can enhance cholinergic function through AChE inhibition [191]. As previously stated, misfolded proteins can trigger the activation of inflammatory mediators such as NF-κB, iNOS, and COX-2, which in turn leads to the upregulation of multiple interleukins and pro-inflammatory cytokines, driving inflammation and, ultimately, neurodegeneration. Within this context, inhibition of these cascades by bioactive nutraceuticals has been reported to exert neuroprotective effects [189]. Figure 2 summarizes the pathogenesis of misfolded protein-mediated neurodegeneration initiated by their activation (modified from Markkal et al. [189]).

**Figure 3:**
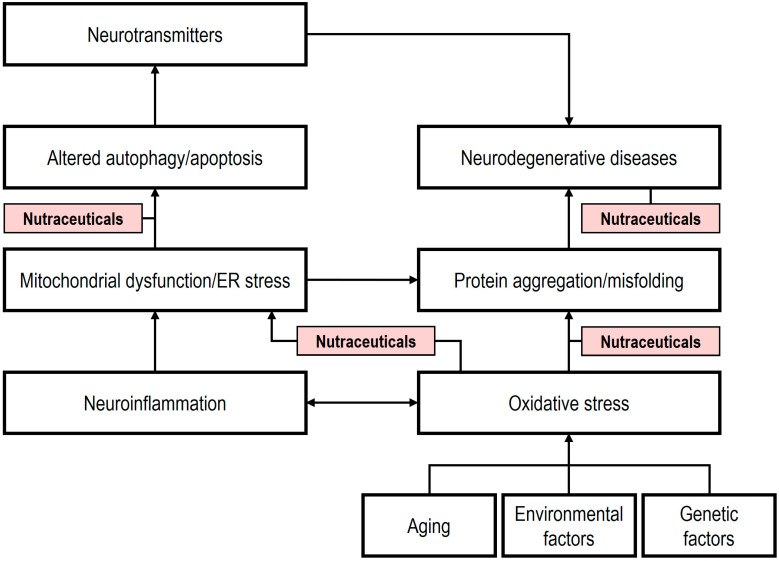
Mechanisms of nutraceuticals in neurodegenerative diseases. Rectangles in red: inhibitory effects. Nutraceuticals exert inhibitory effects on several pathological processes, including (i) modulation of autophagy and apoptosis through restoration of mitochondrial homeostasis and reduction of endoplasmic reticulum (ER) stress; (ii) prevention of protein aggregation and misfolding; and (iii) mitigation of oxidative stress. Conversely, nutraceuticals promote neurogenesis by enhancing the proliferation of neural progenitor cells, as well as the expression of growth factors and neurotrophins (modified from [192]).

The pathophysiology of many neurodegenerative diseases involves multiple factors, including neuro-oxidative stress, inflammation, mitochondrial dysfunction, calcium overload, autophagy, elevated endoplasmic reticulum stress, and apoptosis [192]. Current pharmacological interventions for these diseases fall into three main classes: (i) cholinesterase inhibitors, (ii) NMDA receptor antagonists, and (iii) dopamine replacement therapy [193]. Cholinesterase inhibitors, such as donepezil, galantamine, and rivastigmine, enhance cholinergic transmission by acting on the cholinergic system of the brain. NMDA receptor antagonists, including memantine, mitigate excitotoxic damage by modulating glutamate activity. Dopamine replacement therapy, employing drugs such as levodopa and dopamine agonists, remains a cornerstone in managing the motor symptoms of PD by replenishing dopamine levels in the brain. In contrast, the mechanisms of nutraceuticals capable of crossing the BBB are distinct. These compounds exhibit potent antioxidant, anti-inflammatory, anti-protein aggregation, and anti-apoptotic properties [192]. In addition, they modulate abnormal mitochondrial dynamics, highlighting their potential as effective therapeutic agents against neurodegenerative diseases, with the goal of slowing neuronal degeneration [194]. Figure 3 illustrates the mechanisms of nutraceuticals involved in neurodegenerative disease modulation.

Regarding human studies, a limited number of randomized clinical trials have assessed cognitive outcomes following phytonutraceutical administration. Although relatively few in number, these studies provide important insights into how such compounds may influence brain function, reflecting a growing interest in understanding the therapeutic potential of dietary interventions in promoting cognitive health. Most of these trials were conducted in elderly populations with AD or PD, a group particularly vulnerable to cognitive decline in which even modest improvements in mental performance or mood could substantially enhance quality of life. Table 3 summarizes several clinical trials investigating the therapeutic potential of various nutraceuticals in neurodegenerative disorders and related conditions.

Omega-3 fatty acids show strong mechanistic plausibility, but translation into therapeutic benefit remains uncertain. Key challenges include small and heterogeneous samples, short intervention periods, lack of standardized outcomes, and methodological inconsistencies that limit comparability. The absence of clear dose–response data and the difficulty of disentangling nutrient interactions further complicate interpretation. These gaps highlight the need to clarify whether omega-3 exerts meaningful effects on brain health or simply reflects context-dependent roles. Curcumin shows mechanistic promise, but translation into cognitive or clinical benefit also remains uncertain. Limitations include poor bioavailability, lack of temporal resolution in biomarker changes, small and heterogeneous samples, and short intervention periods that obscure long-term effects. Potential subgroup-specific responses and even adverse cognitive signals are also aspects that should be taken into consideration. Clarifying dose, bioavailability, and links to AD-related pathology is completely essential before drawing firm conclusions. Interpretation of Ginkgo studies is limited by various methodological weaknesses, such as the absence of placebo or blinded designs, monocentric and retrospective approaches, small and selective samples, and reliance on heterogeneous or culturally specific outcome measures. Selection bias and underrepresentation of severe cases constrain generalizability, and imbalances in treatment dosing complicate comparisons with standard therapies. Overall, current evidence remains exploratory, and clarification requires well-controlled, adequately powered trials directly comparing EGb 761 with established agents and in combination regimens. In turn, current findings on ginseng and its derivatives suggest potential neuroprotective effects through antioxidant, anti-apoptotic, and cytoskeletal pathways. However, mechanistic interpretations remain limited. For instance, rapamycin’s lack of specificity prevents firm conclusions on autophagy, protective effects in cell models may not translate in vivo, and candidate biomarkers require validation. Thus, the evidence is preliminary, and rigorous studies are needed to clarify mechanisms and therapeutic relevance in neurodegeneration.

## 5. Novel Nutraceuticals: Mechanisms and Therapeutic Potential

Recent studies have explored novel applications of food-based medicines, particularly for mitigating cognitive decline in AD and symptoms of depression. The growing global elderly population has contributed to a rising prevalence of these conditions, imposing a significant social and economic burden. A substantial body of research has also examined the potential of herbal supplements to alleviate adverse effects associated with medical treatments such as radiotherapy and chemotherapy. In recent years, novel nutraceuticals have emerged, including astaxanthin (AX), cannabidiol (CBD), monk fruit, and Nigella seeds [224].

### 5.1. Astaxanthin

AX is a xanthophyll carotenoid naturally produced by the microalga *Haematococcus pluvialis*, the yeast *Pfaffia rhodozyma*, and the bacterium *Paracoccus carotinifaciens*. AX is a derivative of ascorbic acid (vitamin C) and has been extensively studied for its biological effects [225]. Moreover, it is the pigment responsible for the pink to red coloration of seafood such as salmon, trout, lobster, crab, shrimp, and fish roe.

The biological effects of AX have been attributed to its strong antioxidant activity, modulation of PPARs, and regulation of signaling pathways involved in inflammation and apoptosis [226,227]. In this respect, AX enhances PPARα activity while suppressing PPARβ/δ and PPARγ. In addition, through retinoid X receptor-mediated regulation of PPARα and PPARγ, it helps maintain lipid homeostasis and prevent hepatic injury. AX exhibits cardioprotective, neuroprotective, and retinoprotective effects that are thought to be partly mediated by PPAR modulation [226]. AX also inhibits NF-κB-dependent signaling, reducing the expression of inflammatory mediators such as IL-1β, IL-6, and TNF-α. Depending on the context, AX can exert either anti-apoptotic or pro-apoptotic effects by altering apoptotic protein expression and mitochondrial apoptosis pathways [227]. Clinically, AX has been investigated for the prevention and treatment of atherosclerosis, cancer chemoprevention, and a range of dermatological conditions [228,229]. Furthermore, AX is also used as a sports supplement to improve performance and recovery [230]. Its safety in humans is supported by preclinical studies showing no liver or other pathological changes in experimental animals [231].

### 5.2. Cannabidiol

CBD is the most widely studied non-euphoric phytocannabinoid found in *Cannabis sativa* [232]. Unlike Δ9-tetrahydrocannabinol (THC), which produces euphoric effects via activation of brain cannabinoid CB1 receptors, CBD exhibits very low affinity for both CB1 and CB2 receptors and has no known abuse potential. CBD exerts diverse pharmacological effects, including analgesic, anti-inflammatory, and behavioral modulation, through multiple mechanisms. These include modulation of the endocannabinoid system and transient receptor potential channels, activation of PPARγ and serotonin 1A (5-HT1A) receptors, antagonism of G-protein-coupled receptor 55 (GPR55), and inhibition of adenosine reuptake [233,234].

CBD has gained considerable attention in clinical practice due to its efficacy in managing drug-resistant seizures, particularly in severe early-onset epilepsy, as well as in two rare epileptic syndromes: Dravet syndrome [235] and Lennox–Gastaut syndrome [236]. This clinical evidence led to FDA approval of CBD for treating these conditions in patients aged two years and older. Beyond its anticonvulsant properties, CBD exerts diverse effects in the CNS, including antidepressant effects in humans [234,237]. In addition, it has anxiolytic properties mediated via 5-HT1A receptor activation and can facilitate the extinction of contextual fear memory, potentially through indirect CB1 receptor activation [238]. CBD also modulates mesolimbic dopaminergic activity and influences schizophrenia-related molecular pathways distinct from those targeted by conventional antipsychotics [239]. Analgesic effects of CBD are mediated through activation of 5-HT1A and transient receptor potential cation channels [240], and it has been shown to reduce neuropathic pain in models of chemotherapy-induced, diabetic, chronic constriction, and injury-related neuropathy [240,241,242,243,244]. Furthermore, CBD enhances outcomes in animal models of arthritis through dual immunosuppressive and anti-inflammatory mechanisms, including suppression of cell-mediated and humoral immunity, inhibition of immune cell proliferation, maturation, and migration, as well as modulation of antigen presentation and humoral responses [240,245].

A systematic review assessing the potential benefits of CBD for certain mood-related mental health conditions, including anxiety, BD, depression, psychotic disorders, schizophrenia, and substance use disorders, highlighted significant limitations in the majority of the studies examined [246]. Nevertheless, a recent holistic review emphasized the high potential of cannabinoids such as CBD in the context of mental health, underscoring their promising clinical utility across several conditions, with reported benefits such as improved seizure control in epilepsy and related syndromes and symptomatic relief in AD, dementia, multiple sclerosis, and PD, as well as positive outcomes in ADHD, anxiety-related disorders, ASD, BD, fibromyalgia, migraine, pain-related disorders, post-traumatic stress disorder, schizophrenia, sleep-related disorders, and Tourette and Rett syndromes [247]. These findings are substantiated by other studies, which have reported promising effects of CBD in specific conditions, such as reducing anxiety in particular contexts and managing schizophrenia [248]. Clinically, CBD has also been evaluated for treatment-refractory Crohn’s disease [249] and to alleviate symptoms of ulcerative colitis [250]. In this respect, evidence from clinical trials points to the need for long-term studies to fully assess the risk–benefit profile of CBD.

### 5.3. Monk Fruit

Monk fruit (*Siraitia grosvenorii*), also known as luo han guo, momordica fruit, or longevity fruit, belongs to the *Cucurbitaceae* family. Monk fruit has a long history of use in beverages within the framework of traditional Chinese medicine. Its global popularity is increasing as a low-calorie sweetener in foods and beverages, similar to stevia, with growing interest in its applications for managing certain health-related conditions, such as diabetes and obesity. Monk fruit contains triterpene glycosides, known as mogrosides, which are responsible for its sweet taste and some of its medicinal applications, including the treatment of bronchitis, tonsillitis, cough, and sore throat [224]. The fruit also contains flavonoids, polysaccharides, and essential oils, with certain components demonstrating in vitro anti-asthmatic, antitussive, glucose-lowering, and immunomodulatory effects [251].

Although limited research supports the medical use of monk fruit in clinical contexts, preclinical research has provided some evidence of its biological effects. In mice, mogrosides were shown to attenuate acute lung injury, partly through modulation of the TLR4/MAPK/NF-κB pathway via AMPK activation [252]. Other preclinical studies have confirmed the antioxidant, antimicrobial, and additional pharmacological properties of this fruit [253]. Mogrosides have also demonstrated glucose-lowering effects in murine models of gestational diabetes, mediated by activation of the AMPK signaling pathway [254]. In humans, a study by Tey et al. [255] reported that a beverage containing monk fruit had no significant effect on total daily energy intake, blood glucose, or insulin responses in healthy males. Despite animal toxicity studies being conducted, a formal safety assessment is required for EU registration of monk fruit as a “novel food”.

### 5.4. Nigella Seeds

*Nigella sativa*, commonly known as black caraway, black cumin, or black seed, has been used as both a food and medicine since antiquity, being recognized as a potential therapeutic tool for multiple diseases. For this reason, extensive research has focused on the chemical, biological, and clinical properties of Nigella seeds, investigating applications across a wide range of conditions, including asthma, back pain, bronchitis, chronic headaches, diabetes, dysmenorrhea, fever, gastrointestinal disorders, infertility, inflammation, and obesity [253,256]. External application of the seed oil has been documented for treating abscesses, asthma, atherogenesis, dyslipidemia, eczema, glucose metabolism disorders, nasal ulcers, and swollen joints [224,256]. The primary bioactive compound in Nigella seeds and oil is thymoquinone (TQ, 2-methyl-5-isopropyl-1,4-benzoquinone). The oil also contains linoleic, oleic, and dihomolinoleic acids, EPA, thymol, thymohydroquinone, and dithymoquinone, while the seeds contain trace alkaloids, including nigellidine, nigellicine, and nigellicimine [257].

Engels and Brinkmann [253] identified numerous clinical studies reporting the effects of *N. sativa* seed powder and oil on respiratory diseases, obesity, and inflammatory disorders, including rheumatoid arthritis, dysmenorrhea, mastalgia, and metabolic conditions. Several systematic reviews have evaluated the impact of *N. sativa* preparations on glycemic control, obesity, hypertension, and plasma lipid concentrations [258,259,260,261]. Additional clinical trials have investigated the renal-stone-dissolving properties of Nigella preparations [262], as well as their efficacy in treating ulcerative colitis [263]. The biological activity of these preparations is thought to be primarily attributable to TQ, which has been shown to exert anti-inflammatory and immunomodulatory effects by modulating NF-κB, IL-1β, and TNF-α signaling pathways [257]. TQ has also demonstrated anticancer activity in animal models, likely through alterations in the expression of signal transducer and activator of transcription (*STAT*) genes.

Neuroinflammation has been identified as a major contributing factor in the development of neurodegenerative diseases such as PD and AD, which are associated with increased ROS production. In this respect, TQ has been shown to inhibit the overactivation of NF-κB and its binding to DNA, thereby reducing neuroinflammation. In addition, TQ can inhibit inducible iNOS, resulting in decreased nitrite levels. Thus, the attenuation of iNOS protein expression has emerged as a promising therapeutic strategy for managing inflammatory and autoimmune diseases. TQ also plays a pivotal role in suppressing neuronal damage by attenuating TLR activation and associated inflammatory signaling pathways, thereby reducing the risk of apoptosis and neurotoxicity. Furthermore, TQ has been demonstrated to mitigate glutamate-induced neurotoxicity, prevent mitochondrial dysfunction, and decrease ROS production, collectively protecting neurons from apoptosis. In AD, TQ reduces neurotoxicity induced by Aβ and prevents amyloid plaque accumulation, preserving neuronal integrity. In PD, TQ supports the survival of dopaminergic neurons and alleviates motor symptoms by reducing oxidative stress and safeguarding mitochondria against the MPTP (1-methyl-4-phenyl-1,2,3,6-tetrahydropyridine) toxin. Moreover, TQ enhances spatial memory by protecting neurons from oxidative damage through free radical scavenging. In turn, it has been shown to inhibit AChE, thereby increasing acetylcholine levels, which are crucial for maintaining long-term memory [264]. Observations also suggest that TQ possesses anticonvulsant, antidepressant, anxiolytic, and antipsychotic properties, underscoring its potential for treating drug abuse or addiction, as well as for improving memory and cognitive function [264].

## 6. Gut Microbiome Interactions with Nutraceuticals and Phytochemicals

The human GM is predominantly composed of anaerobic or facultative bacteria, with the phyla Bacillota, Bacteroidota, Pseudomonadota, and Actinomycetota constituting over 90% of the total microbial population [105]. The GM is considered a central component of bidirectional communication between the gut and the brain, known as the GBA [265,266]. This communication occurs through multiple complex pathways that transmit sensory information from the gastrointestinal tract and convert it into hormonal, neural, and immunological signals, which are then relayed to the CNS either individually or in combination [267,268]. Interactions between the microbiota and the GBA are thought to occur via three primary mechanisms [269,270,271,272,273]: (i) direct and indirect signaling through chemical mediators, including microbial metabolites (e.g., SCFAs), hormones, and neurotransmitters, which can be synthesized by or modulated in concentration by the GM [267,274,275,276,277]; (ii) neural pathways, notably through modulation of vagus nerve activity [107,278]; and (iii) immune-mediated signaling, such as microglia-mediated effects and cytokine-driven modulation of hypothalamic–pituitary–adrenal (HPA) axis activity [274,279].

Prebiotics are defined as substrates selectively utilized by host microorganisms, conferring health benefits [280]. These include non-digestible carbohydrates and plant polyphenols. In close relationship with this concept, psychobiotics can be broadly defined as any substance that exerts microbiome-mediated psychological effects or possesses neuroactive properties mediated by the GM, encompassing probiotics, prebiotics, synbiotics, and postbiotics [281,282]. These compounds interact with the colonic microbiota, generating a bidirectional relationship. The GM has substantial enzymatic capacity to degrade plant constituents, producing metabolites with altered bioavailability and pharmacological activity. In addition, plant constituents can modulate the composition and function of the GM, enhancing levels of beneficial bacteria and GM-derived metabolites [283]. Accordingly, the term phytopsychobiotics has been proposed to describe plant-derived nutraceuticals that exert mental health effects mediated via GM modulation, either through prebiotic-mediated effects, postbiotic-mediated effects from GM-derived metabolites of non-digestible plant components, or antibiotic-mediated effects that reduce pathogenic bacterial populations [284].

The term phytochemicals encompasses a diverse range of non-nutritive, plant-derived compounds that have demonstrated protective or disease-preventive properties. They are commonly found in fruits, vegetables, legumes, and grains. Although phytochemicals are nonessential nutrients (i.e., not required by the human organism for survival), they play significant biological roles. Plants naturally produce these compounds as a defense mechanism. However, emerging evidence indicates that they can also confer protective effects in humans against a variety of diseases [285]. To date, over a thousand phytochemicals have been identified, including prominent groups such as carotenoids, dietary fibers, isoprenoids, phytosterols, polyphenols, saponins, and specific polysaccharides [286].

Analogous to the metabolism of drugs of abuse, dietary phytochemicals can be metabolized and transformed by gut microorganisms through processes studied in pharmacomicrobiomics [287]. This field investigates how variations in the microbiome impact the disposition, efficacy, and response to substances and xenobiotics [288]. Specifically, it examines how intra- and inter-individual differences in microbial composition affect bioavailability, drug action, metabolism, pharmacodynamics, pharmacokinetics, potential toxicity, and therapeutic outcomes, rather than focusing on drug–drug interactions with particular microorganisms [287]. A comprehensive understanding of phytochemical metabolism and pharmacokinetics is essential to leverage the therapeutic potential of nutraceuticals in the prevention and treatment of various diseases [289]. The bioavailability of phytochemicals is limited by factors such as individual variations in digestive capacity, membrane transporters, and metabolic enzymes, resulting in only a small fraction being directly absorbed after oral administration. The majority of phytochemical metabolism occurs in the liver and small intestine [290]. In the liver, phytochemicals undergo a first phase of biotransformation reactions, including oxidation, demethylation, and hydrolysis, followed by a second phase of reactions, in which they are conjugated with endogenous molecules to form smaller compounds that enhance absorption. Despite limited absorption in the upper gastrointestinal tract, the GM in the lower gastrointestinal tract can significantly improve phytochemical bioavailability [291]. Microbiota-mediated biotransformation processes, including cleavage, demethylation, dihydroxylation, and deglycosylation, generate metabolites with increased bioavailability and enhanced bioactivity [292,293].

Thus, the relationship between phytochemicals and the human GM constitutes a complex, bidirectional interaction [294]. Phytochemicals can initially modulate the composition of the GM by inhibiting pathogenic microorganisms and promoting the growth of beneficial bacteria. In turn, the GM influences the biotransformation of phytochemicals into their metabolites [295,296]. The fermentation and degradation of microbial metabolites, including SCFAs and other bioactive compounds, not only provide essential substrates and energy for microbial growth but also have the capacity to modulate multiple pathways in the gut, liver, and pancreas, thereby contributing to improvements in enteric health [295]. Overall, in mechanistic terms, phytochemicals and other nutraceuticals interact bidirectionally with the GM, modulating microbial composition by promoting beneficial species and suppressing pathogens. Subsequently, microbial metabolism transforms nutraceuticals into bioactive metabolites with enhanced bioavailability and pharmacological activity. Collectively, these interactions influence neural, immune, and endocrine pathways, ultimately contributing to gut–brain communication, modulation of inflammation, and maintenance of neuronal and metabolic homeostasis. Thus, these integrated processes underscore the GM as both a target and mediator of nutraceutical effects on brain and systemic health.

### 6.1. The Gut Microbiome and Nutraceuticals

#### 6.1.1. Curcumin

Curcumin undergoes extensive metabolism, including reduction, sulfation, and glucuronidation, in the liver, kidneys, and gut mucosa following oral administration [297]. The liver serves as the primary site of curcumin metabolism, although the intestine and GM also contribute significantly to this process. In hepatocytes and enterocytes, the double bonds of curcumin are reduced, producing dihydrocurcumin, hexahydrocurcumin, octahydrocurcumin, and tetrahydrocurcumin [298,299]. The GM can deconjugate second-phase metabolites and convert them back into first-phase metabolites, generating cleavage products such as ferulic acid, which exhibit enhanced bioavailability [300].

The bioactivity of curcumin, similar to that of other dietary polyphenols, depends not only on its absorption but also on its metabolism by the GM. Despite its limited plasma and tissue bioavailability, curcumin demonstrates preferential distribution and accumulation in the gut following oral or intraperitoneal administration [301]. The human GM can metabolize curcumin through multiple pathways, producing active metabolites with both local and systemic effects. These transformations include reduction of the heptadienone backbone and demethylation, processes mediated by bacteria such as *Blautia* spp. [302,303].

The biotransformation of nutraceuticals is mediated by microbial enzymes produced by the GM, and consequently, the efficiency of curcumin metabolism varies according to the microbial profile of the host [304,305]. In the colon, a key contributor to curcumin modification is *Escherichia coli*, which utilizes a nicotinamide adenine dinucleotide phosphate (NADPH)-dependent curcumin/dihydrocurcumin reductase. This enzyme converts curcumin first to dihydrocurcumin and subsequently to tetrahydrocurcumin [306]. The initial step involves reductive cleavage of the chromophoric diarylheptatrienone chain, generating dihydrocurcumin. Multiple gut bacterial species participate in curcumin metabolism. Notably, *Blautia* spp. produce demethylcurcumin and bisdesmethylcurcumin via demethylation [302]. Furthermore, *E. coli* and *Escherichia fergusonii* strains contribute to the formation of dihydrocurcumin, tetrahydrocurcumin, and ferulic acid [307], although the precise roles of these strains require further investigation. Other microorganisms capable of degrading curcumin include *Bifidobacterium longum*, *Bifidobacterium pseudostrandum*, *Enterococcus faecalis*, *Lactobacillus acidophilus*, and *Lacticaseibacillus casei*. Ultra-performance liquid chromatography analysis has identified 23 curcumin-derived metabolites and revealed several novel human GM–curcumin metabolic pathways [308]. Importantly, many curcumin metabolites demonstrate bioactivity equal to or exceeding that of the parent compound. For instance, tetrahydrocurcumin exhibits superior free-radical scavenging activity, conferring therapeutic potential, particularly in the context of neurodegenerative diseases [309].

A substantial proportion of orally administered curcumin escapes digestion in the upper gastrointestinal tract, reaching the colon, where it can exert pharmacological effects by modulating GM diversity and composition [310]. In a clinical study, curcumin treatment was associated with a 69% increase in bacterial species abundance, whereas the control group exhibited a 15% decrease. Treated subjects consistently showed increases in *Bacteroides* spp., *Clostridium* spp., *Citrobacter* spp., *Enterobacter* spp., *Enterococcus* spp., *Klebsiella* spp., and *Pseudomonas* spp., along with a decrease in *Blautia* spp. Using a mouse model, Liu et al. [311] reported significant GM alterations following intravenous curcumin administration, including increased levels of *L. acidophilus*, *Bifidobacterium bifidum*, and *Limosilactobacillus reuteri*, alongside reductions in pathobionts such as *Bacteroides fragilis*, *E. coli*, *Fusobacterium nucleatum*, and *Akkermansia muciniphila*, which correlated with decreased disease severity. Although *A. muciniphila* is generally considered beneficial, its reduction in this study appears to reflect a context-dependent role, in which overabundance during microbial infection can disrupt intestinal mucus, compromise barrier integrity, and trigger inflammation. In rats, curcumin reduced the Bacillota/Bacteroidota ratio and counteracted high-fat-diet-induced GM dysbiosis, effects that were linked to decreased hepatic ectopic fat deposition, lower inflammatory markers, and improved intestinal barrier integrity. These findings suggest that curcumin may represent a novel therapeutic strategy for metabolic disorders, including metabolic dysfunction-associated steatotic liver disease (MASLD) [312]. Moreover, Zhang et al. [313] demonstrated in an ovariectomized rat model that curcumin partially restored GM richness and composition altered by estrogen deficiency, increasing the abundance of *Serratia* spp., *Shewanella* spp., *Pseudomonas* spp., *Papillibacter* spp., and *Exiguobacterium* spp., while reducing *Anaerotruncus* spp. and *Helicobacter pylori* [314].

In addition, curcumin and its derivatives have demonstrated neuroprotective properties by modulating the GBA and reducing gut inflammation via multiple molecular mechanisms [315,316]. In mice, curcumin administration enhanced spatial learning and memory and reduced hippocampal amyloid plaque burden. Notably, curcumin also induced significant changes in the relative abundances of several bacterial taxa, including *Bacteroidaceae*, *Prevotellaceae*, *Lactobacillaceae*, and *Rikenellaceae* at the family level, and *Prevotella*, *Bacteroides*, and *Parabacteroides* at the genus level. Many of these taxa have been implicated in the development of AD. The GM of AD mice also produced eight curcumin metabolites, some of which possess neuroprotective properties [317]. In clinical trials, Baum et al. [206] conducted a placebo-controlled pilot study in Chinese AD patients who received 1 or 4 g of curcumin daily for six months. The results showed a slight improvement in cognitive function, likely due to slower neural decline, although the changes were not statistically significant. Curcumin has also been evaluated in patients with MDD, with treatment for 4–8 weeks producing modest antidepressant effects [318]. Sanmukhani et al. [319] reported that 1000 mg/day of curcumin effectively and safely reduced scores on the Hamilton Depression Rating Scale in MDD patients. Most clinical trials have used curcumin dosages ranging from 0.1 to 1.5 g, with a few studies administering up to 4–5 g. A systematic review by Voulgaropoulou et al. [143] confirmed the beneficial effects of curcumin on neurodegenerative diseases and mental health, particularly AD and depression in humans. Nevertheless, further clinical trials are needed to conclusively determine its neuroprotective efficacy across different neurodegenerative and mental health conditions [192].

#### 6.1.2. Ginseng

The pharmacological effects of ginseng are strongly associated with the GM, which serves as the primary mediator of ginsenoside conversion [320,321,322,323]. Thus, the GM plays a crucial role in enhancing the bioavailability of nonpolar ginsenosides through biotransformation [324]. This process can be further modulated by gut bacteria, such as *Bacteroides*, *Bifidobacterium*, and *Eubacterium* [325]. To date, a total of 289 ginsenosides, including free ginsenosides, have been identified, with their biological activity expected to improve significantly following microbial transformation [326]. In addition, the administration of extracted ginsenosides has been shown to consistently enhance the production of SCFAs, suggesting that saponins may preferentially promote the growth of SCFA-producing bacteria [327,328]. SCFAs, in turn, play a pivotal role in gut–brain communication by exerting neuroactive effects on signaling pathways involving the immune and endocrine systems [329].

Following oral administration, ginseng or red ginseng ginsenosides are exposed to stomach acid, digestive enzymes, and bacterial enzymes, which facilitate their breakdown and metabolism by the GM. The resulting metabolites are subsequently absorbed from the intestine into the bloodstream [330]. Ginseng has been shown to positively modulate several bacterial taxa associated with intestinal and neurological health, including *Akkermansia*, *Bacteroides*, *Bifidobacterium*, and *Lactobacillus*, while concurrently reducing pathogenic bacteria such as *Helicobacter* and members of the phylum Deferribacterota. In rats, the ingestion of ginseng extract over 34 weeks led to a decrease in members of the family *Bifidobacteriaceae* and the genus *Lactobacillus*, as well as to an increase in the phylum Pseudomonadota, the family *Methylobacteriaceae*, and the genera *Parasutterella* and *Sutterella* [331]. Notably, *Lactobacillus mucosae* and *B. longum* have been shown to synergistically alleviate stress-induced anxiety and depression in mice by mitigating GM dysbiosis [332]. Prolonged administration of ginseng extract in rats also resulted in a significant increase in beneficial bacteria, including *Allobaculum*, *Bifidobacterium*, *Clostridium*, and *Lactobacillus*, while pathogenic bacteria such as *Alistipes*, *Butyricimonas*, *Helicobacter*, and *Parabacteroides* were significantly reduced. These findings suggest that long-term consumption of ginseng extract may help inhibit the colonization of pathogenic bacteria and promote a healthier GM [333].

The pharmacological benefits of ginseng are largely attributed to natural antioxidant compounds extracted from the berries, leaves, roots, and stems of the plant [321,322,323]. These bioactive compounds also contribute to neurogenesis, neuronal growth, neurotransmission, and synaptogenesis, thereby providing protective effects for the CNS [334]. Treatment with Korean red ginseng (KRG) has been shown to increase the relative abundance of the family *Lachnospiraceae* and the order Clostridiales compared to untreated controls [335]. Moreover, the prebiotic effects of ginseng on beneficial bacteria, including *A. muciniphila*, *Bifidobacterium* spp., and *Lactobacillus* spp., have been documented in multiple studies [336,337]. These effects are associated with the maintenance of intestinal barrier function, as ginseng polysaccharides promote the growth of *Bacteroides* spp. and *Lactobacillus* spp., which play key roles in the metabolism of ginsenosides, the restoration of the GM, and the enhancement of intestinal metabolism and absorption of specific ginsenosides [338].

Ginseng extract, ginsenosides, and ginseng-containing preparations have been shown to exert neuroprotective effects and improve memory disorders through modulation of the GM [339]. Supplementation with *Panax quinquefolius* and *P. notoginseng* saponins has been associated with enhanced cognitive function and improved short-term memory in patients with AD, potentially mediated by GM modulation and upregulation of neurotransmitters [340]. In addition, significant increases in acetate, butyrate, and propionate levels have been observed in AD patients following supplementation, correlating with increased abundances of *Lactobacillus* and *A. muciniphila* [341]. Microbial diversity, which affects multiple metabolic pathways, is typically reduced in AD mice compared to wild-type controls, leading to lower levels of SCFAs, amyloid deposition, cognitive deficits, and intestinal abnormalities [342]. The GM composition in AD patients is distinct and has been identified as a significant risk factor for disease progression [343,344]. Specifically, fecal analyses of AD patients reveal increased levels of Bacteroidota and decreased abundances of Actinomycetota and Bacillota, including reductions in families such as *Clostridiaceae*, *Ruminococcaceae*, and *Turicibacteraceae* within the Bacillota phylum [345]. Therapeutic interventions such as the Qisheng Wan formula, which contains ginseng and ginsenoside Rg1, have been shown to restore disrupted GM and reduce inflammatory markers, mitigating AD symptoms [346,347]. Similarly, administration of KRG at 30 mg/kg/day in a rat AD model led to *Lactobacillus* dominance within the GM, restoration of BBB integrity, reduced microglial activation, and improved memory and cognition, indicating that KRG can alleviate AD pathology via the GBA [321].

PD is a neurodegenerative disorder and the second most common movement disorder. Patients with PD exhibit a range of symptoms, including gait difficulties, bradykinesia, and tremors, often accompanied by behavioral and cognitive impairments [348]. Multiple studies have demonstrated that the GM composition in PD patients is distinct from that of healthy controls and individuals with other neurological disorders [349,350]. Notably, gastrointestinal dysfunction in PD may precede the onset of key neurological symptoms [350,351]. PD patients typically exhibit a depletion of SCFA-producing bacteria from the family *Lachnospiraceae*, which are implicated in maintaining intestinal barrier integrity [352,353]. Compared to healthy controls, the GM of PD patients shows a predominance of the phylum Verrucomicrobiota and the genera *Mucispirillum*, *Lactobacillus*, *Parabacteroides*, and *Porphyromonas*, whereas controls exhibit higher abundances of *Prevotella*, suggesting that dysregulated immune responses may contribute to PD-associated inflammation [354]. Moreover, elevated levels of Verrucomicrobiota have also been implicated in neurodegeneration. In this context, intervention with red ginseng has been shown to reduce Verrucomicrobiota levels significantly, while administration of KRG extract has been associated with a relative increase in *Eubacterium* and a decrease in *Ruminococcus* and Verrucomicrobiota, which may contribute to improvements in PD symptoms and neurological function [351].

#### 6.1.3. *Ginkgo biloba* Extracts

Extracts of Gb leaves are used globally in standardized formulations and contain diterpene lactones (ginkgolides A, B, C, and J), the sesquiterpene lactone bilobalide, flavonoids (primarily as glycosides), and polysaccharides [294]. The flavonoids in Gb extracts are mainly present as glycosides, including kaempferol, quercetin, and isorhamnetin, which are linked to mono-, di-, or triglycosides via β-glycosidic bonds. The major terpene lactones comprise the diterpenes ginkgolides A, B, and C, as well as the sesquiterpene bilobalide. Following oral administration, terpene lactones are absorbed in the gastrointestinal tract in their intact or original form. However, the absorption of intact flavonol glycosides is typically inefficient [355,356,357]. Glycosidases produced by the GM hydrolyze these flavonoid glycosides into absorbable aglycones, which are then absorbed and further conjugated to glucuronides or sulfates by host second-phase enzymes [358,359,360].

The use of Gb for neurological disorders associated with cognitive impairment has been explored in the context of anxiety and depression. In this respect, the composition and function of the GM can be altered in response to disease. When plant-derived compounds are biotransformed by the GM in vivo, changes in the microbial community caused by disease may affect their metabolism and absorption in the digestive tract. Consequently, these modifications could influence the systemic effects of the compounds [361]. A study was conducted to investigate the potential antidepressant mechanisms of Gb [362]. The efficacy of a polysaccharide fraction derived from leaf extract was evaluated in terms of its effects on GM composition and depressive-like behaviors in mice. Compared to the untreated control group, administration of the extract attenuated stress-induced depressive behaviors and ameliorated GM dysbiosis, resulting in increased richness of *Lactobacillaceae*. Moreover, oral administration of *L. reuteri* or fecal microbiota transplantation (FMT) from Gb-treated mice to depressive mice significantly reduced immobility time in the forced swimming test. These results suggest that modulation of the GM by Gb polysaccharides may contribute to the alleviation of depressive symptoms, potentially via the GBA [363]. In addition, this plant extract has been reported to mitigate atherosclerosis [364] and to restore intestinal barrier integrity through the GBA [365]. Nevertheless, the precise mechanisms underlying these effects remain to be fully elucidated. Yu et al. [366] demonstrated that treatment with Gb extract ameliorated memory deficits in APP/PS1 mice and induced significant changes in GM composition and metabolite profiles. Specifically, supplementation with the extract increased the relative abundances of *Bifidobacterium pseudolongum*, *L. reuteri*, *Turicibacter* sp. TS3, *Coriobacteriaceae*, *Adlercreutzia caecimuris*, and *A. muciniphila*, bacterial taxa recently recognized for their health-promoting effects.

#### 6.1.4. *Hypericum perforatum*

A medicinal herb frequently employed for the treatment of depression is *H. perforatum*, widely known as St. John’s wort. A substantial body of research supports the therapeutic potential of this plant in managing mild to moderate depression. Several studies have demonstrated that its efficacy is comparable to that of selective serotonin reuptake inhibitors, while exhibiting a lower incidence of adverse effects and reduced discontinuation risk [367]. *H. perforatum* contains multiple bioactive compound classes implicated in its antidepressant activity, including hyperforins, polyphenols such as flavonoids like hyperoside, naphthodianthrones (hypericin), and procyanidins [294]. In a recent animal study, the impact of *H. perforatum* on GM composition was evaluated in ovariectomized rats. Administration of the extract was shown to reverse ovariectomy-induced alterations in the GM at the phylum level and to attenuate the increase in the Bacillota/Bacteroidota ratio associated with estrogen deficiency [368].

According to Zhang et al. [369], hyperforin exerted a pronounced effect on the fecal metabolite profiles of stressed mice, inducing significant alterations in 239 metabolites. These metabolites were primarily associated with co-metabolism pathways and microbiota-specific processes. Notably, carbocysteine was identified as a key metabolite positively correlated with beneficial bacteria, including *A. muciniphila* and *Muribaculum intestinale*, and its levels were significantly elevated following hyperforin administration. Behavioral assessments further demonstrated that carbocysteine supplementation ameliorated depressive-like behaviors in the chronic restraint stress mouse model. Furthermore, carbocysteine was shown to enhance colonic mucus production and strengthen mucus barrier integrity.

In another study, the administration of *H. perforatum* effectively reversed depression-like behaviors in rats subjected to chronic unpredictable mild stress [370]. This intervention was associated with a significant reduction in corticosterone levels and the adrenal index, alongside a marked increase in serotonin concentrations. Alpha and beta diversity analyses of the GM revealed enhanced richness and a higher relative abundance of members belonging to the phyla Actinomycetota and Bacillota. Moreover, increases were observed in the families *Erysipelotrichaceae*, *Bifidobacteriaceae*, and *Atopobiaceae* and the genera *Dubosiella* and *Bifidobacterium*. In contrast, the relative abundances of the phyla Pseudomonadota, Euryarchaeota, Mycoplasmatota, and Elusimicrobiota; the families *Ruminococcaceae*, *Christensenellaceae*, and *Lachnospiraceae*; and the genus *Eubacterium* were decreased. Linear discriminant analysis identified Lactobacillales (order), *Lactobacillaceae* (family), *Limosilactobacillus*-related (genus), and *L. reuteri* (species) as key biomarkers in the *H. perforatum* group, which were also predicted to modulate metabolic pathways involved in carbohydrate and amino acid metabolism, as well as in signal transduction.

#### 6.1.5. *Lavandula* spp. Essential Oils

Lavender (*L. angustifolia*) oil has been traditionally employed for the management of sleep disturbances, transient insomnia, mental stress, and mood disorders [371]. Research has demonstrated that essential oils derived from *Lavandula* spp. possess antibacterial activity, showing efficacy against multiple serotypes of *E. coli* and *Salmonella enterica* subsp. *enterica* serovar Newport [372]. Beyond its antibacterial properties, lavender essential oil has also exhibited anti-parasitic effects [373,374]. In this respect, Baker et al. [375] investigated the therapeutic potential of essential oil from a novel lavender cultivar, *Lavandula intermedia* cv. Okanagan lavender (OLEO), in a mouse model of acute colitis induced by *Citrobacter rodentium*. Oral administration of OLEO significantly attenuated disease severity, as evidenced by reductions in morbidity and mortality, intestinal tissue damage, and infiltration of neutrophils and macrophages. Treatment with OLEO also decreased levels of pro-inflammatory mediators, including TNF-α, IFN-γ, IL-22, macrophage inflammatory protein-2α, and inducible nitric oxide synthase. Furthermore, OLEO supplementation modulated the GM, promoting enrichment of Bacillota members, which are associated with protection against *C. rodentium*-induced damage, while decreasing the abundance of Pseudomonadota.

In another study, the inclusion of dry *L. angustifolia* leaves in the diet of rats resulted in a decrease in typical *E. coli* populations and a concurrent increase in the weakly fermenting form of this bacterium [376]. Dietary supplementation with *L. angustifolia* also led to a significant reduction in populations of *Enterococcus* spp., *Proteus* spp., and *Pseudomonas* spp., accompanied by an increase in *Staphylococcus aureus*, *S. epidermidis*, and *Candida albicans*. In a subsequent study, Li et al. [377] examined the effects of lavender essential oil inhalation on GM composition, metabolite profiles, and hippocampal gene expression in alcohol-withdrawn depressive rats. Using 16S rDNA sequencing analysis, the authors observed a marked decrease in Bacteroidota and a significant increase in members of the family *Muribaculaceae* within the gastrointestinal tract of these animals. Moreover, inhalation of lavender essential oil significantly attenuated hippocampal levels of pro-inflammatory cytokines, including IL-2, IL-6, IL-1β, and TNF-α.

### 6.2. The Gut Microbiome and Phytochemicals

#### 6.2.1. Polyunsaturated Omega Fatty Acids

Over an extended period of time, research has indicated that omega-3 polyunsaturated fatty acids (omega-3 PUFAs) confer benefits across cardiovascular, cognitive, immune, mental, and metabolic health domains. Increasing evidence suggests a bidirectional relationship between omega-3 PUFAs and the GM, with omega-3 PUFAs capable of modulating GM diversity and abundance, and with the GM, in turn, capable of influencing the metabolism and absorption of these fatty acids. Notably, an imbalanced intake of omega-3 to omega-6 PUFAs has been associated with GM dysbiosis, characterized by a marked increase in the Bacillota/Bacteroidota ratio, which may contribute to disease development [378].

The literature on omega-6 PUFAs has yielded inconsistent results compared to omega-3 PUFAs, particularly regarding their role in promoting GM eubiosis. A study by Liu et al. [379] further explored this issue by investigating the effects of plant-derived omega-6 and omega-3 PUFAs in a double-blind, randomized trial involving 51 patients with marginal hyperlipidemia. During the 12-week intervention, participants consumed 4 g of omega-3-rich oil (41.2% comprising α-linolenic acid and stearidonic acid) and 4 g of corn oil (44.4% omega-6). The omega-3 group exhibited a significant reduction in total cholesterol levels. In addition, an increase in the relative abundance of Bacteroidota, along with a significant decrease in Bacillota, was observed, resulting in a cumulative reduction of the Bacillota/Bacteroidota ratio. Although healthy controls were not included in this study, the findings reinforced the superior effect of omega-3 PUFAs compared to omega-6. At the genus level, omega-3 supplementation led to a notable reduction in *Phascolarctobacterium* and *Veillonella*. Furthermore, monounsaturated fatty acids have been shown to exert a beneficial effect on the GM. This was corroborated by Anavi-Cohen et al. [380], who assessed the impact of a high-oleic peanut cultivar (rich in oleic acid) compared to a standard peanut cultivar (low in oleic acid) in healthy C57BL/6J mice over 18 weeks. Although metabolic outcomes were not substantially altered, the high-oleic diet promoted an increase in the prevalence of *Bifidobacterium*, *Coprococcus*, and *Lactobacillus* genera.

Omega-3 PUFAs have been shown to exert a multifaceted influence on the GM through three primary mechanisms: (i) modulation of the composition and relative abundance of gut microorganisms; (ii) regulation of pro-inflammatory mediators, including endotoxins (LPS) and IL-17; and (iii) modulation of SCFAs or their salts [381]. These fatty acids can promote a beneficial GM profile by inhibiting the growth of *Enterobacteriaceae* and enhancing *Bifidobacterium* populations, thereby mitigating the inflammatory responses associated with metabolic endotoxemia [382]. In a case report examining the effects of an omega-3 PUFA-rich diet on the human GM, a significant increase in several butyrate-producing genera, including *Blautia*, *Bacteroides*, *Coprococcus*, and *Roseburia*, was observed [383]. Moreover, omega-3 PUFAs have been reported to stimulate the proliferation of beneficial bacteria such as *Akkermansia*, improve the intestinal microenvironment, increase mucosal thickness, reinforce intestinal barrier integrity, and facilitate weight reduction via the regulation of genes involved in lipid metabolism [384].

As previously noted, omega-3 PUFAs are capable of directly modulating the composition of the GM, while the GM can influence the absorption, bioavailability, and biotransformation of these fatty acids, thereby affecting the balance of PUFA intake and function. Dietary omega-3 PUFAs are partially metabolized by anaerobic bacteria, including *Bifidobacterium* and *Lactobacillus*, in the distal intestine, which can alter the distribution of intestinal microbial communities in both animal models and humans [385,386]. Specific microbial species, such as *Proteus* spp. (formerly *Bacillus proteus*), *Butyrivibrio fibrisolvens*, *Clostridium proteoclasticum*, *Myxococcus xanthus*, and *Lactiplantibacillus plantarum*, have been shown to convert the omega-3/omega-6 PUFA precursors α-linolenic acid and linoleic acid into conjugated α-linolenic acid and conjugated linoleic acid, respectively [381,387]. These compounds may subsequently undergo hydrogenation to form saturated fatty acids, such as stearic acid (C18:0), thereby reducing the overall PUFA content [388]. The influence of gut microorganisms on omega-3 PUFA metabolism and absorption is likely mediated, at least in part, by SCFAs. Supplementation with omega-3 PUFAs has been demonstrated to reversibly increase the abundance of SCFA-producing bacteria, including *Bifidobacterium*, *Lactobacillus*, and *Roseburia*, within the intestinal tract of mice [389].

In contrast, omega-3 PUFAs have been reported to directly modulate the abundance of mucolytic bacteria with detrimental effects. These bacteria constitute a distinct group of microorganisms residing within the mucus layer, utilizing mucin as an energy source [390]. Pathogenic mucolytic bacteria have been shown to degrade the mucin layer and alter its viscoelastic properties, a process that may be influenced by changes in mucus pH. Such degradation compromises the protective mucus barrier, facilitating bacterial colonization of the gut epithelium and potentially leading to infection [391]. Conversely, commensal mucolytic bacteria can compete with pathogens for attachment sites [392] and, in some cases, promote mucus production and increase layer thickness [393,394]. Following omega-3 PUFA treatment, the relative abundances of potentially pathogenic mucolytic bacteria, including *Ruminococcus* spp., *Dorea longicatena*, *Oscillibacter* spp., and *Eubacterium hallii*, were significantly reduced, whereas the commensal mucolytic bacterium *A. muciniphila* exhibited a corresponding increase in relative abundance [395].

Omega-6 PUFAs constitute another important class of fatty acids, primarily encompassing linoleic acid (18:2 omega-6 PUFA) and arachidonic acid (20:4 omega-6 PUFA). Few studies have specifically addressed the effects of omega-6 PUFAs on the GM, as oils rich in omega-6 are often employed as control or placebo interventions in omega-3 studies. Current literature presents a divergence of findings regarding the relative influence of omega-6 and omega-3 PUFAs on GM regulation. In a *C. rodentium*-induced colitis mouse model, Ghosh et al. [396] reported that omega-3 supplementation resulted in a significant increase in fecal *Lactobacillus* and *Bifidobacterium* levels, coupled with a reduction in gut inflammation, compared to the omega-6 group. Similarly, in a separate study, mice receiving tuna oil, which is rich in omega-3 fatty acids, exhibited changes in the relative abundances of *Turicibacter* and *Akkermansia* genera compared with those receiving soybean oil, which is rich in omega-6 fatty acids. Moreover, serotonin and its metabolites in the amygdala were elevated in the tuna oil group, suggesting a potential modulation of the GM, immune system, and brain function [397].

Besides having direct, opposing effects on the GM, omega-6 PUFAs can indirectly attenuate the beneficial actions of omega-3 PUFAs through metabolic competition. Both omega-6 and omega-3 PUFAs are incorporated competitively into the phospholipid bilayer of cell membranes, resulting in distinct modifications in membrane fluidity, permeability, and stability [398]. In addition, omega-6 and omega-3 PUFAs utilize the same set of metabolic enzymes but elicit different pro-inflammatory biological effects via competitive binding to certain enzyme groups [399,400]. Consequently, elevated omega-6 levels can directly influence omega-3 metabolism, thereby modulating its beneficial effects.

#### 6.2.2. Anthocyanins

Anthocyanins are a type of flavonoid naturally present as pigments in a wide variety of plant-based foods [401,402]. This group includes compounds such as pelargonidin, cyanidin, delphinidin, peonidin, petunidin, and malvidin, all of which possess a phenolic structure that underlies their biological activities, including improved vascular function, cancer prevention, and enhancement of bone health [403]. Studies have shown that anthocyanins can delay or inhibit the onset of certain diseases, largely due to their anti-inflammatory and antioxidant properties [404]. Phenolic compounds can suppress pro-inflammatory mediators, thereby mitigating inflammation. Furthermore, anthocyanin metabolites have been reported to activate the Nrf2/HO-1 pathway, enhancing antioxidant enzyme activity and related defense mechanisms. They may also reduce intestinal inflammation by modulating the MAPK and NF-κB pathways, mediated via TAK1 and SphK/S1P signaling [402].

The bioavailability of natural anthocyanins has been estimated to be as low as 2% [405]. During metabolism, only a small fraction of anthocyanins is absorbed by the gastrointestinal tract and transported to various tissues, including the liver and kidneys. The majority, however, bypasses the small intestine and reaches the colon, where it undergoes hydrolysis and fermentation by the GM, facilitating further digestion [406]. Colonic metabolites of anthocyanins are subsequently transported to the liver for processing and distribution throughout the circulatory system, contributing to diverse biological effects. Although only a limited portion of dietary anthocyanins is absorbed directly, the majority that reaches the colon interacts with key microbial genera such as *Bifidobacterium* spp. and *Lactobacillus* spp., which exhibit glucosidase activity. During growth, these microorganisms can metabolize phenolic compounds, generating energy that supports the proliferation of other gut bacteria [289]. These bacterial groups are associated with beneficial effects in the large intestine, including inhibition of pathogenic microbes through SCFA production and competition for substrates and adhesion sites [401]. In vitro studies conducted by Tian et al. [407] revealed that the bacterial metabolism of anthocyanins includes the cleavage of glycosidic bonds, breakdown of anthocyanidin rings, and conversion into phloroglucinol derivatives and benzoic acids. Probiotic strains such as *L. plantarum* and *Streptococcus thermophilus* have been shown to efficiently degrade cyanidin-3-glucoside and cyanidin-3-rutinoside, respectively, representing the maximal conversion of these compounds by gut bacteria [408].

Anthocyanin consumption can modulate GM colonization, thereby affecting the growth and activity of intestinal bacteria. Several studies have highlighted the beneficial roles of genera such as *Akkermansia*, *Bifidobacterium*, and *Lactobacillus* in promoting host health. These bacteria have been reported to metabolize anthocyanins in cooperation with *Bacteroides* and *Eubacterium* species [409]. In addition to the advantageous effects in the large intestine [401], these bacteria can reduce populations of potentially harmful species, such as *Hathewaya histolytica* (previously *Clostridium histolyticum*), which has been associated with tumorigenesis and inflammatory bowel disease [410]. In mouse models of colon cancer, oral administration of bilberry anthocyanin extract increased microbial diversity in the gut, along with higher abundances of *Clostridium* spp. and *Lactobacillus johnsonii*. Daily supplementation with bilberry anthocyanins also inhibited tumor growth and improved responses to immunotherapy [411]. In vitro experiments by Faria et al. [401] demonstrated that malvidin-3-glucoside enhanced the growth of *Bifidobacterium* spp. and *Lactobacillus* spp. when incubated with fecal microbiota, while *Bacteroides* spp. remained unaffected. Interestingly, combining malvidin-3-glucoside with other anthocyanins produced a synergistic effect, further promoting bacterial growth.

Hidalgo et al. [412] reported that mixtures of anthocyanins stimulated the growth of *Bifidobacterium*, *Lactobacillus*, and *Enterococcus* in a batch fermentation model designed to simulate the human distal colon. Notably, the microbial metabolite malvidin-3-glucoside demonstrated similar beneficial effects to the original anthocyanin compounds [405]. In another study, Liu et al. [413] observed that oral administration of anthocyanins to C57BL/6J mice significantly counteracted high-fat-diet-induced weight gain, reducing body weight by 20–27%. The treatment also decreased total adipose tissue mass by 18–25% and lowered plasma total cholesterol levels by approximately 25%. These changes were accompanied by a substantial reduction in plasma LPS concentration, which correlated with decreased abundances of *Rikenella* and members of the *Rikenellaceae* family. Furthermore, supplementation with berry-derived anthocyanin extracts promoted the proliferation of *Lachnoclostridium*, *Roseburia*, and *Clostridium innocuum*, leading to enhanced fecal SCFA production [413]. Across multiple studies, anthocyanins consistently appeared to alleviate high-fat-diet-induced dysbiosis by reducing populations of *Ruminococcus* and *Muribaculaceae* while supporting an increase in *Oscillibacter* [413,414].

The neuroprotective potential of anthocyanins is partly linked to their ability to modulate the GM [415]. Marques et al. [414] explored this relationship in rats subjected to a high-fat diet, which is known to induce obesity-related neuroinflammation and behavioral disturbances through GM alterations. Supplementation with anthocyanin-rich blackberry extract led to increased *Oscillibacter* abundance in the gut. These findings suggest that the gastrointestinal effects of anthocyanins contribute to their anti-neuroinflammatory properties, partly by downregulating TCK-1 expression. In addition, anthocyanins may influence CNS function by affecting tryptophan metabolism through the kynurenine pathway, promoting the formation of neuroprotective metabolites while concurrently reducing systemic inflammation [414]. Multiple studies have shown that anthocyanins can suppress pro-inflammatory cytokine expression, thereby mitigating neuroinflammation and potentially preventing AD pathogenesis [416]. Specifically, black soybean anthocyanins have been reported to protect against AD and preserve synaptic function in the Aβ1-42 mouse model [417]. Similarly, anthocyanins derived from Korean black beans reduced neurodegeneration in APP/PS1 transgenic mice by lowering ROS levels and inhibiting apoptosis via the PI3K/Akt/GSK3 signaling pathway, which activates the Nrf2/HO-1 cascade and its downstream targets [418]. Moreover, anthocyanin-loaded polyethylene glycol–gold nanoparticles have been shown to modulate the p-PI3K/p-Akt/p-GSK3β axis in the Aβ1-42 mouse model, preventing tau hyperphosphorylation at serines 413 and 404 [419].

#### 6.2.3. Quercetins

Quercetin, which constitutes a polyphenolic flavonoid, is found naturally in a variety of plant sources and is also present in certain beverages. Its antioxidant activity plays a key role in the prevention and management of numerous conditions, including osteoporosis, cancer, and cardiovascular disorders [420,421,422]. Human pharmacokinetic studies have indicated that quercetin exhibits relatively low bioavailability following a single oral dose, due to limited absorption, rapid metabolism, and/or swift excretion [422]. Once absorbed, quercetin is transported to the liver, where it undergoes first-phase and second-phase metabolic transformations, yielding metabolites such as quercetin-3-sulfate and quercetin-3-glucuronide, which circulate through the bloodstream and reach various tissues [423]. Quercetin that escapes absorption in the small intestine enters the large intestine, where it is metabolized by colonic microbiota into phenolic acids, which can then be absorbed and transported to the liver via the portal vein circulation [424].

Quercetin exerts a substantial influence in shaping the gut environment, thereby influencing the regulation of the GM. On one hand, quercetin is metabolized by gut microorganisms, and the resulting metabolites may possess distinct biological activities compared to the original compound. On the other hand, quercetin has been shown to alter the composition of the GM, fostering the growth of beneficial microorganisms while inhibiting potentially harmful species. This dual capability underlies the therapeutic potential of quercetin through modulation of the GM, ultimately promoting host health [425]. Within the intestine, quercetin is transformed by microbial activity into compounds such as 3,4-dihydroxyphenylacetic acid (also called 3-[3-hydroxyphenyl] propionic acid), 3,4-dihydroxybenzoic acid, and 4-hydroxybenzoic acid. Bacterial taxa involved in quercetin metabolism include *B. fragilis*, *Clostridium perfringens*, *Streptococcus* spp., *Lactobacillus* spp., *Bifidobacterium* spp., and *Eubacterium* spp. [426,427]. In a seminal study, Lan et al. [428] reported that quercetin supplementation significantly enhanced microbiota diversity, with marked changes observed in genera such as *Clostridium*, *Bacteroides*, and *Bacillus*. Notably, quercetin intake increased *Lactobacillus* abundance while reducing *Ruminococcus*. Similarly, Shi et al. [429] demonstrated that quercetin improved GM diversity in antibiotic-treated mice, highlighting its potential as a prebiotic intervention for managing GM dysbiosis.

#### 6.2.4. Catechins

Catechins, which belong to the polyphenol family, are present in a wide range of dietary sources [430,431]. The main catechins include epigallocatechin, epicatechin, epicatechin-3-gallate, and epigallocatechin-3-gallate, with the latter being the most abundant and biologically active [432,433]. Their chemical structures, particularly the catechol and pyrogallol groups, are responsible for their potent antioxidant capacity and overall bioactivity [434]. Research has highlighted the health-promoting properties of catechins, which encompass anti-inflammatory, antimicrobial, immunomodulatory, and neuroprotective effects [435].

The bioavailability of catechins in humans is notably low. After oral intake, only a small fraction of catechins becomes systemically available [436], as they are transported to the liver through the portal vein circulation. In the liver, second-phase metabolic enzymes convert these compounds into methylated, glucuronidated, and sulfated derivatives. It is estimated that approximately two-thirds of ingested catechins pass to the colon, where microbial enzymes degrade them into a variety of metabolites [427,437]. These microbial products can then enter the enterohepatic circulation or the systemic bloodstream, exerting multiple physiological effects [438]. The GM plays a pivotal role in catechin biotransformation through several pathways: (i) hydrolysis of galloyl esters by microbial esterases; (ii) opening of the C-ring; and (iii) modifications via lactonization, decarboxylation, dehydroxylation, and oxidation reactions [439,440]. These processes generate smaller molecules such as phenylvalerolactones and phenylvaleric acids [437], which may be absorbed through the colonic epithelium into systemic circulation [441]. In this context, Kutschera et al. [442] identified *Flavonifractor plautii* and *Eggerthella lenta* as two bacterial strains capable of converting dietary catechins into hydroxyvaleric acid and valerolactone metabolites.

Multiple in vitro studies have suggested that catechins may act as prebiotic agents by selectively promoting the growth of beneficial gut microorganisms [443,444]. Research has shown that green tea catechins can stimulate populations of advantageous bacteria, such as *Bifidobacterium* spp. and *Lactobacillus* spp., while simultaneously inhibiting the proliferation of potentially harmful microorganisms, including *Clostridium* spp., in controlled fermentation experiments. Catechin consumption has also been linked to increased GM diversity and a lower Bacillota/Bacteroidota ratio in various animal models [445,446]. In murine studies, Liao et al. [447] observed that tea catechins significantly promoted bifidobacterial growth while reducing serum total cholesterol and LDL cholesterol levels. Moreover, dietary catechins were found to enhance the relative abundance of *A. muciniphila*, contributing to alleviation of high-fat-diet-induced metabolic syndrome [448]. In rats, combining catechins with a high fructooligosaccharide diet resulted in decreased body weight, accompanied by increases in *Parabacteroides*, *Phascolarctobacterium*, *Robinsoniella*, and *Prevotella*, alongside reductions in *Lachnospira*, *Clostridiales*, *Ruminococcus*, *Peptococcaceae*, and *Oscillospira* [449].

#### 6.2.5. Chlorogenic Acid

Chlorogenic acid (CGA) is a prevalent dietary polyphenol present in many fruits (e.g., apples, blueberries), vegetables (e.g., artichokes, green coffee beans, potatoes), and herbs such as sage. As a hydroxycinnamic acid-derived phenolic compound, CGA possesses multiple health-promoting properties [450,451]. Research has highlighted its potential benefits, including antioxidant, anti-inflammatory, anticancer, and neuroprotective effects [450].

The hydrophilic nature of CGA limits its ability to traverse lipophilic membranes following oral intake, resulting in low absorption and poor bioavailability [452]. Research indicates that CGA undergoes distinct absorption and metabolic processes across the gastrointestinal tract, liver, and kidneys. Its metabolic pathways can be summarized as follows: (i) approximately one-third of dietary CGA is absorbed intact into the bloodstream without prior hydrolysis in the stomach or upper gastrointestinal tract; (ii) a small fraction (about 7%) is absorbed in the small intestine, partially hydrolyzed into caffeic and quinic acids; (iii) the majority reaches the colon, where GM-mediated metabolism produces various metabolites that are subsequently absorbed; and (iv) both intact CGA and its microbially degraded products can undergo further absorption and metabolism in the liver [453,454]. Studies in rat models indicate that CGA experiences minimal hydrolysis in the stomach and exhibits negligible bioavailability before reaching the colon [455]. Once in the large intestine, CGA is extensively broken down by the GM into low-molecular-weight aromatic acids, including metacoumaric acid and derivatives of phenylpropionic and benzoic acids, which are thought to mediate most of the biological effects of CGA [456,457,458]. Interestingly, the transformation of caffeoylquinic acid has been shown to be enhanced in the presence of *Bifidobacterium animalis* subsp. *lactis* [459].

CGA has been shown to modulate the relative abundances of certain microbial taxa, including members of the phylum Bacillota, the orders Burkholderiales and Desulfovibrionales, the *Clostridium coccoides*–*Eubacterium rectale* group, and the genera *Desulfovibrio*, *Klebsiella*, and *Bifidobacterium*, which are considered beneficial to the host [452,455,456]. Conversely, dietary CGA supplementation has been associated with a reduction in *Bacteroides* populations in ileal samples from weaned pigs [457], as well as with decreased abundance of Pseudomonadota in cecal samples [460].

Ye et al. [461] explored the role of the GM in mediating the protective effects of CGA against obesity and metabolic endotoxemia in C57BL/6 mice. Supplementation with CGA resulted in shifts in microbial composition, notably increasing the abundance of SCFA-producing bacteria such as *Dubosiella*, *Romboutsia*, *Mucispirillum*, and *Faecalibaculum*, as well as *Akkermansia*, which was linked to improved intestinal barrier integrity. In a separate study, Wang et al. [462] reported that six weeks of CGA administration led to a marked reduction in *Desulfovibrio*, *Lachnospira*, and *Ruminococcus*, while promoting the growth of Bacteroidota members and lactobacilli. Shi et al. [463] investigated the effects of CGA in a high-fat-diet-induced mouse model of non-alcoholic fatty liver disease, finding that CGA alleviated hepatic steatosis and inflammation, decreased serum transaminases, and improved insulin sensitivity. In addition, CGA supplementation increased *Bifidobacterium* levels in feces and simultaneously reduced *E. coli* abundance.

## 7. Discussion

The aim of this review was to synthesize recent evidence on nutraceuticals, particularly in relation to their effects on mental health and brain function. Based on the literature examined, several key points can be highlighted: (i) Essential fatty acids, prebiotics, and phytochemicals from various foods contribute to mood regulation, cognitive function, and overall mental health via metabolic, anti-inflammatory, and GBA mechanisms. (ii) Probiotics support neurological and mental health by modulating the GM, immune responses, and metabolic pathways. (iii) Nutrients such as amino acids, vitamins, minerals, and omega-3 fatty acids are linked to improved mental health, alleviation of depressive and anxiety symptoms, and neuroprotection. (iv) GM modulation via dietary fiber and SCFA production influences brain function, cognition, and neuroinflammation, impacting disorders such as AD, BD, depression, and PD. (v) Omega-3 PUFAs are essential for neuronal membrane integrity, neurotransmission, and neurogenesis, with deficiencies linked to conditions such as ADHD, ASD, BD, dementia, depression, and schizophrenia. (vi) Hyperforin and other *Hypericum* compounds exert neuroprotective, anti-inflammatory, antioxidant, and anti-apoptotic effects, potentially reducing depression, excitotoxicity, and AD-related pathology. (vii) Curcumin enhances serotonin and dopamine release while inhibiting glutamate, exerting antidepressant and anxiolytic effects, and also reduces inflammation and oxidative stress, promoting cognitive function. (viii) Silexan reduces anxiety and depressive symptoms by modulating serotonergic signaling, inhibiting T- and N-type calcium channels, and enhancing neuroplasticity via PKA/CREB and MAPK pathways. (ix) Gb extract EGb 761 improves cognitive function and neuroprotection by reducing oxidative stress, inflammation, and apoptosis through its flavonoids, terpenoids, and bilobalide. (x) Ginseng compounds protect against stress and sleep deprivation-induced cognitive deficits, support neuroplasticity, and exhibit neuroprotective effects relevant for AD and age-related memory decline. (xi) Nutraceuticals can prevent or slow neurodegeneration by targeting misfolded proteins and modulating associated pathways, including oxidative stress, neuroinflammation, mitochondrial dysfunction, and dysregulated signaling. (xii) AX exerts neuroprotective effects primarily through strong antioxidant activity, modulation of PPAR signaling, and inhibition of NF-κB-dependent inflammation, reducing IL-1β, IL-6, and TNF-α. (xiii) CBD exerts neuroprotective, anticonvulsant, anti-inflammatory, analgesic, anxiolytic, and antidepressant effects through multiple mechanisms, demonstrating efficacy for drug-resistant epilepsy and showing promise for a range of conditions, such as mood-related disorders, neuropathic pain, neurodegenerative diseases, and schizophrenia. (xiv) Monk fruit contains triterpene glycosides, flavonoids, polysaccharides, and essential oils, which exhibit antioxidant, antimicrobial, immunomodulatory, anti-asthmatic, and glucose-lowering effects in preclinical studies. (xv) Nigella seeds contain bioactive compounds, primarily TQ, along with fatty acids, thymol, and alkaloids, that exhibit anti-inflammatory, immunomodulatory, and anticancer effects through modulation of NF-κB, IL-1β, TNF-α, and STAT signaling pathways. (xvi) phytochemicals act as phytopsychobiotics, influencing mental health through GM modulation, as they can alter microbial composition, inhibit pathogens, promote beneficial bacteria, and be biotransformed by the GM into metabolites with enhanced bioavailability and bioactivity, which confers therapeutic effects on cognition, mood, and gut–liver–pancreas health. (xvii) Curcumin modulates the GBA by altering GM composition, as it enhances beneficial bacteria and reduces pathobionts, thereby providing neuroprotective and antidepressant effects. (xviii) Ginseng modulates the GBA by promoting beneficial bacteria and SCFA production, enhancing gut barrier integrity, neurotransmitter regulation, and neuroprotection. (xix) Ginsenosides and ginseng extracts improve cognitive and neurodegenerative outcomes, including memory enhancement and reduced AD and PD pathology, likely via GM-mediated biotransformation, anti-inflammatory effects, and synaptic/neurogenic support. (xx) Gb polysaccharides and metabolites alter GM composition, reduce depressive-like behaviors, and enhance memory in preclinical models. (xxi) *H. perforatum* administration enhances fecal metabolites linked to GM co-metabolism, strengthens intestinal barrier integrity, and modulates neurotransmitters such as serotonin, collectively alleviating depressive-like behaviors in preclinical models. (xxii) Lavender essential oil mitigates depressive-like behaviors and stress by modulating the GM, increasing *Muribaculaceae*, and decreasing pro-inflammatory cytokines in the hippocampus. (xxiii) Omega-3 PUFAs improve brain function and mental health by modulating the GM, increasing SCFA-producing and commensal mucolytic bacteria (e.g., *Akkermansia*), reducing pro-inflammatory mediators, and enhancing serotonin metabolism in the amygdala. (xxiv) Omega-6 PUFAs, however, may counteract these benefits through metabolic competition and pro-inflammatory effects. (xxv) Anthocyanins exert neuroprotective effects by modulating the GM, promoting beneficial bacteria and SCFA production, reducing pro-inflammatory cytokines, and influencing tryptophan metabolism via the kynurenine pathway. (xxvi) Quercetin is metabolized into bioactive phenolic acids that increase beneficial bacteria such as *Lactobacillus*, which may support neuroprotection and cognitive function. (xxvii) Catechins are metabolized into bioactive phenolic metabolites that increase beneficial bacteria such as *Bifidobacterium* and *Lactobacillus*, which contribute to neuroprotection and potentially improve cognitive and metabolic health. (xxviii) Chlorogenic acid is metabolized into bioactive compounds that enhance SCFA-producing and beneficial bacteria, supporting neuroprotection, metabolic health, and intestinal barrier integrity.

**Figure 4:**
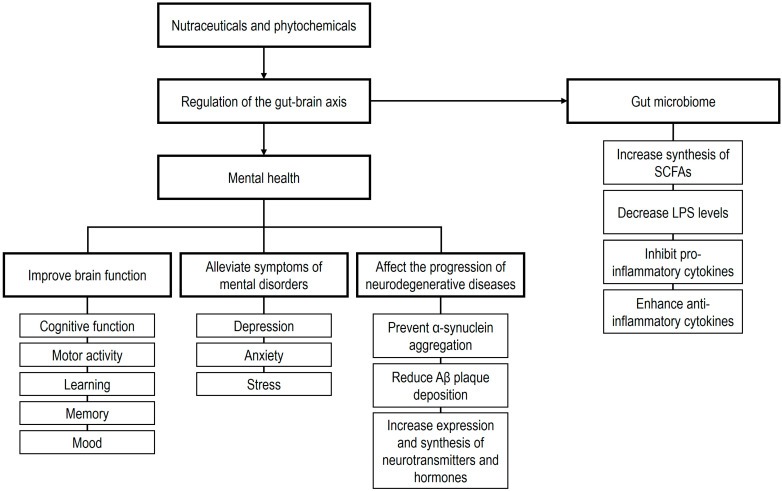
Influence of nutraceuticals and phytochemicals on the GBA and their beneficial effects on mental disorders, neurodegenerative diseases, and GM homeostasis. SCFAs: short-chain fatty acids; LPSs: lipopolysaccharides; Aβ: amyloid-β.

Nutraceuticals hold considerable promise in the field of nutritional psychiatry, as empirical evidence indicates that, when administered at therapeutic doses, they can exert beneficial effects on mental health. Various functional ingredients and phytochemicals have been shown to modulate fundamental physiological, immunological, and neurological processes, thereby facilitating the management of mental disorders and representing key components of nutritional psychiatry [464]. Although their role as a primary treatment modality is not established, nutraceuticals are frequently used in conjunction with conventional pharmaceuticals to provide supportive effects [465]. Greater emphasis on empirical research is essential, particularly regarding plant-derived ingredients such as astaxanthin, cannabidiol, monk fruit, and *N. sativa*, which may accelerate recovery while minimizing adverse effects [244]. Thus, interest in nutraceuticals that promote mental health is steadily increasing [466,467], and as this field evolves, future research may enable the development of customized formulations for specific mental health conditions, explore their potential as alternatives or adjuncts to prescription medications, and investigate the mechanisms by which nutraceuticals and phytochemicals influence mental health [84,468]. Furthermore, the bioactive components of nutraceuticals in dietary foods may help maintain GBA health, given the direct influence of diet on this bidirectional system [469]. Indeed, the bidirectional relationship between the GM and ingested substances underscores how dietary and nutraceutical interventions can modulate microbial composition, thereby impacting gut–brain communication, host behavior, and neurological function [470]. Figure 4 illustrates the beneficial effects of nutraceuticals and their active components on mental health and on the GM (modified from Song et al. [471]).

The inflammatory component associated with neurodegeneration, along with the role of the GM in regulating neurotransmission, reinforces the plausible association of psychiatric and neurodegenerative disorders, including AD, PD, multiple sclerosis, amyotrophic lateral sclerosis, and Huntington’s disease, with GM alterations [472,473]. Epidemiological data suggest that diet may play a preventive or delaying role in prion and prion-like neurodegenerative diseases [474], and diet has been recognized as a major determinant of GM modulation [475]. However, the strongest evidence linking neurodegeneration and GM comes from studies on AD and PD. In AD, altered GM composition has been associated with increased LPS production, disruption of the BBB, and deregulation of Treg/Th2 responses, all of which promote AD development and amyloid-β (Aβ) fibrillogenesis [476,477]. Moreover, amyloid proteins may be released by GM components, as observations indicate that bacteria such as *E. coli*, *S. enterica* subsp. *enterica* serovar Typhimurium, *Bacillus subtilis*, *Mycobacterium tuberculosis*, and *S. aureus* can generate functional amyloid, contributing to the accumulation of misfolded protein aggregates, oligomers, and fibrils [478,479].

In patients diagnosed with PD, motor system impairment has been linked to the presence of non-motor symptoms, which are associated with autonomic dysfunctions now acknowledged as integral aspects of the disease. Among these autonomic dysfunctions, intestinal constipation has been reported to appear up to 20 years prior to the onset of motor symptoms and has been associated with GM alterations [480]. A notable increase in the relative abundance of *Enterobacteriaceae* has been correlated with postural instability [349]. In addition, a decrease in *Prevotella* has been linked to reduced mucin synthesis and increased gut permeability in PD patients [349]. These changes also coincide with alterations in the microbial metabolism of tryptophan and β-glucuronides [481], which are involved in neurotransmitter biosynthesis. Furthermore, GM dysbiosis may contribute to PD pathogenesis by promoting alpha-synuclein aggregation in intestinal submucosal neurons, potentially propagating to the CNS via the vagal nerve pathway [482,483].

The mechanisms by which nutraceuticals interact with the GBA and, subsequently, influence mental health include (i) direct or indirect modulation of GM composition, impacting GM dysbiosis and microbial processes such as the production of microbial metabolites and the regulation of immune responses, and (ii) microbial biotransformation of nutraceuticals, which enhances the bioavailability and bioactivity of their bioactive components. One approach to improve the efficiency of their metabolism and absorption is to administer nutraceuticals via optimized delivery systems [484]. For instance, Chen et al. [485] proposed a novel nano-formulation technique involving the co-encapsulation of epigallocatechin-3-gallate and quercetin in particle-stabilized emulsion gels, which has been shown to enhance the transport and bioaccessibility of these bioactive compounds.

Although previous studies have explored the effects of nutraceuticals on GM composition, limited knowledge exists regarding the synergistic interactions between intestinal epithelial integrity and microbiota in modulating the bioavailability and bioactivity of these compounds. Moreover, most research has relied on fecal samples, which may not accurately represent the small intestinal or cecal microbiome. To achieve a more comprehensive understanding of the dynamic changes occurring in the GM and the intestine, the development of appropriate animal models is essential. For instance, Sun et al. [317] examined the interplay between curcumin and the GM in APP/PS1 double transgenic mice as a model for AD. Their results indicated that curcumin could influence GM diversity, and various metabolites were biotransformed by the microbiota, providing novel insights into potential AD treatments. Addressing these aspects will enable a clearer understanding of the relationship between phytonutrients and the GM, as well as of the mechanisms through which the GM affects the host, thereby informing the future application of nutraceuticals.

Nutraceuticals act as prebiotics by modulating the composition of the GM and by stimulating the growth of beneficial gut microorganisms, potentially conferring a range of health benefits. These effects may include the maintenance of intestinal barrier integrity, enhancement of cognitive functions, and a reduction in the risk of gastrointestinal and neurodegenerative disorders [324]. Regular consumption of nutraceuticals has been shown to influence GM diversity, promoting the proliferation of probiotic bacteria such as *Bifidobacterium* (phylum Actinomycetota), *Lactobacillaceae* (phylum Bacillota), and *Akkermansia* (phylum Verrucomicrobiota). Concurrently, nutraceuticals can reduce the abundance of pathogenic bacterial genera, including *Clostridium*, *Ruminococcus*, *Dorea*, and *Eubacterium* (phylum Bacillota), *Alistipes*, *Bacteroides*, and *Prevotella* (phylum Bacteroidota), as well as multiple genera within the phylum Pseudomonadota. These observations suggest that nutraceuticals may contribute to the prevention of gut-related and neurological disorders. A comprehensive understanding of these interactions is critical for the development of innovative therapeutic strategies that employ the synergistic potential of nutraceuticals and the microbiome. Nevertheless, further studies are required to fully establish their efficacy and safety as complementary interventions for complex health conditions.

Although functional foods and nutraceuticals offer notable health advantages, they also present a number of challenges that must be carefully addressed [486]. Properly managing these issues is crucial to ensure both the safety and effectiveness of these products in promoting overall health and wellness. A key challenge relates to the regulatory landscape of nutraceuticals. The broad spectrum of these products, encompassing fortified foods and dietary supplements, highlights the need for clear guidelines and regulatory frameworks to ensure their safety and effectiveness [487]. Ensuring product quality and standardization constitutes another significant challenge, as variability in functional foods and nutraceuticals can result from factors such as cultivation techniques, processing procedures, and storage conditions. Implementing and upholding industry-wide quality regulations is therefore critical to ensure that consumers receive products offering reliable and reproducible health benefits [488]. In addition, the potential for interactions with medications emphasizes the need for cautious and well-informed use. Bioactive compounds in nutraceuticals may alter the absorption or metabolism of drugs, necessitating collaborative decision-making between healthcare professionals and consumers regarding dietary choices [489]. Individual responses to functional foods and nutraceuticals can vary substantially, influenced by factors including genetics, age, and underlying health conditions [490]. Consequently, adopting personalized nutritional and supplementation strategies is essential to maximize the potential benefits of these products. It is important to further emphasize that plant-derived nutraceuticals, although generally considered safe, can also pose risks of adverse effects when consumed in high doses or from impure sources. A major concern is the potential for interactions with hepatic enzymes, notably the CYP450 system, which can alter the metabolism, absorption, or elimination of co-administered drugs [491]. Although evidence of direct toxicity is limited, certain metabolites, such as those derived from the catechols in green tea extract that are typically attributed to antioxidant activity, may paradoxically promote oxidative stress and have been linked to hepatotoxicity [492]. Similarly, *G. biloba* has been associated with adverse outcomes, including insomnia, dizziness, and interactions with anticoagulants. Antioxidants, conventionally viewed as protective against ROS, may under specific conditions exhibit pro-oxidant behavior. In the presence of reduced metals such as iron or copper, flavonoids and other antioxidant compounds can generate ROS rather than neutralize them. This dual activity is context-dependent, influenced by factors such as dose, environmental conditions, and metal ion availability. Indeed, supraphysiological doses of beta-carotene or vitamin E have been reported to exacerbate oxidative damage, particularly when administered prior to exposure to oxidative stressors such as radiation or cigarette smoke [493].

Within the framework of this review, the role of plant-based diets deserves particular attention; appropriately designed, these dietary patterns can incorporate nutraceuticals and may provide substantial therapeutic potential. In fact, the feasibility of implementing vegetarian diets in hospital settings has been increasingly recognized, with multiple dietary protocols demonstrating positive clinical and economic outcomes, highlighting their capacity to improve patient care and promote sustainability [494]. Vegetarian diets have been associated with a range of health benefits due to their antioxidant, anti-inflammatory, immunomodulatory, anti-proliferative, and anti-hypertensive properties. In addition, dietary components promote microbial synthesis of SCFAs and postbiotics, such as equol, urolithin, enterolignans, isothiocyanates, coprostanol, and secondary bile acids, which exert multiple physiological and immunological effects [495]. Although strict vegetarian diets, such as vegan patterns, may lead to specific nutrient deficiencies, targeted nutritional interventions have proven effective in mitigating these risks. For instance, oral zinc supplementation has successfully restored IRF3 and IFNα levels in zinc-deficient vegetarian and vegan individuals [496]. Regarding mental health, evidence linking vegetarian diets to outcomes such as depression, anxiety, and stress remains heterogeneous, reflecting variability in intervention type, geographic context, and outcome measures [497]. Many studies are also limited by biased participant selection, reliance on subjective data, small sample sizes, unclear intervention duration, and insufficient differentiation between vegetarian subtypes, which complicates reproducibility and interpretation. Furthermore, adherence and follow-up periods are often not reported, and the broad spectrum of dietary categorizations, including vegan and lacto-ovo-vegetarian, restricts direct comparisons [497]. Ultimately, the near future will likely necessitate the widespread adoption of plant-based dietary patterns, driven by the urgent imperatives of environmental sustainability, ethical considerations, and social development [494]. In this context, nutraceuticals emerge as a pivotal tool, offering the potential to address specific nutritional needs while complementing the well-established broad-spectrum benefits of plant-based diets.

Current evidence points to the cognitive benefits of nutraceuticals. Nevertheless, direct causal relationships between nutraceutical supplementation, neurogenesis, and cognitive health remain to be definitively established. Future research should aim to elucidate the molecular targets specific to each nutraceutical and determine the optimal dosing of active compounds to maximize efficacy [498]. In order to promote healthy aging and improve cognitive performance, a range of interventions combining dietary strategies, physical exercise, cognitive stimulation, and social engagement have been applied [499]. Although these multi-domain interventions demonstrate promising outcomes, particularly in terms of quality of life, their heterogeneity and context-dependency limit applicability and reproducibility [499]. Moreover, cognitive stimulation, which is one of the most widely employed non-pharmacological approaches for neurodegenerative conditions, often exhibits limited efficacy, highlighting the need for complementary strategies [3]. For instance, GM-targeted interventions, including psychobiotics and FMT, have emerged as potential adjuvant therapies, showing promising results [500]. Diet, in turn, represents a highly versatile and valuable approach, as it not only confers intrinsic benefits but also naturally accommodates integration with other interventions, such as GM modulation and nutraceutical administration, emphasizing the pivotal role of diet in promoting health and preventing disease, as underscored by several recent studies [501,502,503,504,505]. Thus, properly studied and applied, bioactive compounds from nutraceuticals may provide targeted support for neurodegenerative conditions such as AD and PD, thereby enhancing the clinical relevance of dietary interventions. Furthermore, dietary patterns can induce epigenetic modifications via endocrine and immune pathways, with significant implications for longevity. In this respect, the GM can influence brain cell activity through histone and DNA modifications, acting as an epigenetic effector of host gene expression during aging, although mechanistic insights into these molecular pathways remain limited [506]. Building on this, dietary interventions that modulate epigenetic aging, such as caloric restriction, have been investigated as potential geroprotective strategies, showing modest but measurable effects on DNA methylation-based aging clocks [507,508]. Taken together, these findings underscore the potential of integrative dietary strategies, including nutraceutical supplementation, as a non-invasive approach to enhance brain health and mitigate neurodegenerative risk. Advancing mechanistic understanding and optimizing customized interventions will be essential to fully leverage these benefits in aging populations.

Building on the evidence summarized in this review, it is plausible to state that nutraceuticals exert multifaceted effects on mental health and brain function, acting through complementary mechanisms that involve both direct neurobiological modulation and indirect GM-mediated pathways. Essential nutrients, phytochemicals, and bioactive compounds not only support neurotransmission, neuroplasticity, and neuroprotection but also influence GBA signaling, immune responses, and metabolic pathways, collectively contributing to the prevention or mitigation of neuropsychiatric and neurodegenerative conditions. Moreover, emerging data highlight the role of microbiota-mediated biotransformation in enhancing the bioavailability and efficacy of nutraceuticals, underscoring the potential for diet-based interventions to modulate cognitive outcomes. These findings suggest that future strategies integrating targeted nutraceuticals with GM modulation could optimize mental health benefits and offer novel complementary approaches to conventional therapies.

This review may present several potential limitations that should be properly acknowledged. First, a formal assessment of the quality of the included studies was not performed. Second, the review is narrative in nature, and the search and selection process did not follow a standardized methodology. Third, many of the reviewed studies, although RCTs, exhibit inherent limitations such as small sample sizes, cross-sectional designs, and short intervention durations. Fourth, the review does not explore in depth the underlying mechanisms through which the interventions affect health outcomes. Fifth, there is an insufficient number of studies evaluating the efficacy of novel nutraceuticals, except for CBD, in relation to mental health and brain function. Finally, there is a scarcity of customized formulations capable of establishing causal relationships between nutraceuticals and neuropsychiatric conditions.

## 8. Conclusions

The landscape of functional nutraceuticals offers a diverse range of dietary options with considerable potential to promote health and prevent disease. Scientific evidence supporting these benefits continues to grow, underscoring the importance of incorporating these bioactive compounds into a balanced diet. Of particular interest is the emerging focus on nutraceutical ingredients that may enhance mental well-being. As research in this area progresses, several future considerations merit attention: (i) the expanding body of evidence may enable the development of customized formulations aimed at specific neurodegenerative and psychiatric conditions; (ii) nutraceuticals could potentially serve as alternatives or complements to prescription medications for mental health disorders; and (iii) further studies may elucidate the underlying mechanisms through which these bioactive compounds influence mental health.

Nutraceuticals are absorbed orally and undergo metabolic processes that generate biologically active substances, which subsequently modulate the abundance and composition of the GM. Although research on the interplay between nutraceuticals and microbiota has expanded over the past two decades, it remains in its early stages. In the future, nutraceuticals may be utilized even during pregnancy to support maternal gut health, promote neurodevelopment, and enhance overall infant wellness. Similarly, their application in children could aid in the development of the GM and help prevent adverse conditions, thereby preserving health during adolescence. Moreover, the regular intake of phytonutrients in adults has the potential to provide both preventive and therapeutic health benefits.

Despite the wide range of beneficial effects demonstrated by nutraceuticals, further research is required to elucidate how the GM may enhance these bioactivities and also to deepen the understanding of phytochemical–GM interactions. A promising approach for future investigation is the exploration of the still-unknown potential benefits of nutraceuticals and their microbial metabolites in the prevention and management of specific health conditions, particularly those of neuropsychiatric, neurodegenerative, and psychological origin. Nevertheless, important challenges remain before the aforementioned insights can be effectively translated into clinical and public health applications. Translational gaps persist, as much of the evidence derives from preclinical studies or short-term trials with limited generalizability. Furthermore, the absence of standardized regulatory frameworks and clear guidelines for nutraceutical formulations continues to hinder their clinical integration. Therefore, priority should be given to well-designed, long-term clinical studies that clarify safety, optimal dosing, and efficacy across diverse populations. Without any doubt, these steps will be pivotal to advance the field from promising experimental findings toward validated and widely applicable therapeutic strategies. To date, emerging evidence indicates that the effects of nutraceuticals on mental health may be mediated through complex interactions with the GM, metabolic pathways, and neuroprotective mechanisms, emphasizing their potential for targeted interventions.

## Figures and Tables

**Table 1:** Bioactive components and human health effects associated with nutraceuticals and functional foods.

Functional Foods	Bioactive Components	Nutraceutical/Functional Effects	References
Nutraceutical beverages	- Catechins (epigallocatechin-3-gallate and epicatechin gallate). - Alkaloids (caffeine).	- Improve digestion, enhance overall health, strengthen immunity, improve heart health, and increase energy levels. - Tea is a rich source of bioactive compounds and nutrients with significant antioxidant, anticancer, anti-inflammatory, immune-modulating, antihistamine, antimicrobial, antidepressant, cardioprotective, blood pressure-lowering, nervous system protective, and health-promoting properties. - The ingestion of coffee has a potential impact on the prevalence of various chronic conditions, including but not limited to mental health issues, liver disease, and elevated suicide risk.	[21,22,23,24]
Fermented foods	- Polyphenols. - Dietary fiber. - Probiotics and prebiotics. - Conjugated linoleic acid. - Vitamins (cobalamin). - Fatty acids.	- Immune-modulatory, antioxidant, chemoprotective, anti-inflammatory, cardioprotective, immune-boosting, anticancer, and antidiabetic properties. - The microorganisms used in the fermentation process can enhance the bioavailability of nutrients and augment the bioactive compounds present in food products. - Citrus-blended vinegars can be used as functional foods due to their anti-obesity, anti-aging, and antioxidant properties.	[25,26,27]
Fruits	- Polyphenols and alkaloids. - Vitamins and minerals. - Dietary fiber. - Tannins (ellagitannins, prodelphinidins). - Catechins (gallocatechins). - Anthocyanidins (pelargonidin, cyanidin, delphinidin).	- Antioxidant, cardioprotective, anti-inflammatory, antimicrobial, antidepressive, anticancer, immunomodulatory, nervous system-enhancing, antidiabetic, and anti-obesity properties. - Fruits contain various components, such as antioxidants and polyphenols, which reduce the effect of hormones that are associated with brain disorders.	[28,29,30]
Herbs and spices	- Curcumin. - Gingerol saponin and shogaols. - Dietary fiber. - Alkaloids (piperine). - Capsaicin. - Allicin.	- Antioxidant, antidiabetic, nutritional, cardioprotective, anti-hypercholesterolemic, antimicrobial, anti-inflammatory, anticancer, antihistamine, and blood pressure-lowering properties.	[31,32,33]
Honey and derivatives	- Organic acids. - Royalisin (defensin-1). - Hydrogen peroxide, methylglyoxal, eugenol, and p-coumaric acid. - Flavonoids (naringin, luteolin, chrysin, kaempferol, quercetin, galangin, apigenin, isorhamnetin, pinocembrin, pinobanksin). - Phenolic compounds (syringic acid, gallic acid, ferulic acid, caffeic acid).	- Enhance resistance to infections, alleviate cold symptoms, potentially extend lifespan, promote wound healing, and prevent certain diseases. - Possess antioxidant, anticancer, antidiabetic, anti-inflammatory, immune-boosting, blood pressure-lowering, antihistamine, antimicrobial, cognitive, cardioprotective, and nervous-system-improving properties. - Honey is topically used as an antibiotic in herbal and traditional medicine for the treatment of burns and skin injuries. Honey might play a beneficial role in the management of adverse effects associated with chemotherapy and radiation therapy used in cancer treatment regimes and for the treatment of osteoarthritis.	[34,35,36,37]
Legumes	- Dietary fiber and minerals. - Protein and peptides. - Isoflavones (genistein, daidzein, glycitein). - Phytosterols. - Saponins. - Resveratrol.	- Beneficial role in the prevention and treatment of a wide range of health conditions, including cancer, obesity, cardiovascular diseases, overweight, diabetes mellitus, digestive diseases, and nervous system disorders. - Regular consumption of soybeans can ameliorate menopause symptoms and can reduce the risk of certain types of cancer, such as breast and prostate cancers.	[38,39,40]
Nuts	- α-linolenic acid and polyunsaturated fatty acids. - Minerals and amino acids (L-arginine). - Dietary fiber. - Antioxidants (polyphenols, tocopherols). - Vitamins (folate).	- Synergistic and beneficial effect on vascular and metabolic pathways, improving cardiovascular prognosis and reducing the risk of cardiovascular and cancer diseases. - The ingestion of nuts also promotes moderate improvements in inflammation, endothelial function, blood pressure, and glycemic control, ameliorating and preventing the effects of diabetes, brain disorders, and obesity/overweight.	[41,42,43]
Seeds	- Dietary fiber and essential oils. - Omega-3 fats, polyunsaturated and monounsaturated fats. - Antioxidants (p-coumaric acid). - Phenolic compounds (ferulic acid, catechins, sesamin, caffeic acid, lignans, pinoresinol, sesamolin, lariciresinol). - Saponins (triterpenoid glycosides). - Phytosterols (stigmasterol, sitosterol, secoisolariciresinol diglucoside, campesterol). - Vitamins and minerals. - Triterpenes. - Cyanogenic glycosides (neolinustatin).	- Reduce blood pressure, control blood sugar, decrease LDL cholesterol, reduce the risk of cancer and cardiovascular diseases, and enhance antimicrobial activity. - Polyphenols contained in seeds possess antioxidant, anti-inflammatory, anticancer, immune-promoting, and cardioprotective properties.	[44,45,46]
Vegetables	- Omega-3 fatty acids. - Dietary fiber and oligoelements (zinc, selenium). - Vitamins. - Carotenoids (β-carotene, lycopene). - Xanthophylls (astaxanthin, zeaxanthin, canthaxanthin, cryptoxanthin). - Phenolic compounds (isoflavones, flavonoids). - Catechins (epigallocatechin-3-gallate, epicatechin, epigallocatechin, and epicatechin gallate). - Glucosinolates. - Phytosterols.	- Prevent numerous diseases in humans, including diabetes, cardiovascular diseases, and microbial infections. - The regular inclusion of vegetables in diets results in a decrease in the incidence of cardiovascular diseases, stroke, and cancer.	[28,47,48,49]
Whole grains	- Dietary fiber (β-glucans) and minerals. - Vitamins (folate, vitamin E, tocols). - Prebiotics (inulin). - Phenolic compounds (polyphenols, lignans). - Carotenoids (lutein, zeaxanthin, β-cryptoxanthin, β-carotene). - Amino acids (betaine). - Phytosterols.	- Reduce the risk of diseases such as gastrointestinal disorders, cognitive decline, obesity, cardiovascular diseases, type 2 diabetes mellitus, coronary heart disease, cancer, stroke, and diverticulosis.	[50,51,52]

LDL: low-density lipoprotein.

**Table 3:** Clinical trials on the effects of various nutraceuticals on neurodegenerative disorders.

Nutraceuticals	References	Intervention	Main Results
Omega-3 fatty acids			
	Da Silva et al. [195]	*N* = 31 PD and MDD patients (mean age 64.4 years). 480 mg/day DHA + 720 mg/day EPA. 12 weeks.	Treatment had no statistically significant effect on the rate of change on the Hoehn–Yahr Scale score, but there was a significant decrease in MADRS and CGI scores.
	De Waal et al. [196]	*N* = 179 mild AD patients (≥50 years). 1700 mg/day DHA + 6 mg/day EPA. 24 weeks.	The administration contributed to the maintenance of the organization of brain networks in mild AD patients.
	Eriksdotter et al. [197]	*N* = 174 AD patients (74 ± 9 years). 1720 mg/day DHA + 600 mg/day EPA 24 weeks.	The daily supplementation stabilized the cognitive performance of AD subjects, assessed by ADAS-cog and MMSE scores.
	Jernerén et al. [198]	*N* = 171 AD patients. 1720 mg/day DHA + 600 mg/day EPA. 24 weeks.	The effect of omega-3 supplementation on MMSE and CDR appeared to be influenced by homocysteine plasma levels.
	Olde Rikkert et al. [199]	*N* = 201 patients with mild AD. 1200 mg/day DHA + 300 mg/day EPA (Souvenaid). 24 weeks.	The intake of Souvenaid was well tolerated with a favorable safety profile. The adherence to Souvenaid was very high, reflecting its good tolerability and ease of use.
	Philips et al. [200]	*N* = 42 AD patients (mean age 71.1 years). 625 mg/day DHA + 600 mg/day EPA. 16 weeks.	The daily supplementation was associated with none or only negligible benefits on mood and cognition, assessed by MMSE, HVLT-R, BASDEC, and BADLS.
	Pomponi et al. [201]	*N* = 24 PD patients. 800 mg/day DHA + 290 mg/day EPA. 24 weeks.	No statistically significant effect on the rate of change on either the UPDRS or Hoehn–Yahr Scale score. In DHA-treated patients, the HDRS score was reduced by at least 50%.
	Quinn et al. [202]	*N* = 295 AD patients (mean age 76 years). 2000 mg/day DHA from seaweed. 18 months.	Supplementation with DHA compared with placebo did not slow the rate of cognitive and functional decline in patients with mild to moderate AD assessed by MMSE, ADAS-cog, CDR, ADS-ADL, and NPI.
	Shinton et al. [203]	*N* = 39 AD patients (>55 years). 675 mg/day DHA + 975 mg/day EPA. 675 mg/day DHA + 975 mg/day EPA + 600 mg/day LA. 12 months.	Active groups were not different from the placebo group in ADAS-cog. The omega-3 + LA group showed less decline assessed by MMSE.
	Taghizadeh et al. [204]	*N* = 60 PD patients. 1000 mg omega-3 fatty acids + 400 IU vitamin E. 12 weeks.	Treatment had favorable effects on the UPDRS score.
	Wang et al. [205]	*N* = 15 AD patients (mean age 72.5 years). 1720 mg/day DHA + 600 mg/day EPA. 24 weeks.	A decrease was observed in RvD1 and LXA_4_ production from peripheral blood mononuclear cells of AD patients who did not receive omega-3, but not in cells of AD subjects under omega-3 intake.
Curcumin			
	Baum et al. [206]	*N* = 27 AD patients (>50 years). Dose CUR 1–3 g. 24 weeks.	No differences in cognitive decline were observed.
	Cox et al. [207]	*N* = 60 healthy elderly patients (60–85 years). Dietary Longvida with 400 mg/kg of CUR. 4 weeks.	Single-dose improved performance on working memory and sustained attention.
	Rainey-Smith et al. [208]	*N* = 96 healthy elderly people (40–90 years). Dose: CUR 1500 mg. 12 months.	Cognitive decline after 6 months in the placebo group, but not in the curcumin group.
	Ringman et al. [209]	*N* = 30 mild-to-moderate AD patients (>49 years). Dose: CUR 4 g. 24–48 weeks.	Non-significant change on MMSE scores after CUR administration. No alterations of AD biomarkers.
	Small et al. [210]	*N* = 40 non-demented subjects (51–84 years). Dose: CUR 90 mg. 18 months.	CUR improved memory and attention performance and prevented neuropathological deposition in the amygdala and hypothalamus.
*Ginkgo biloba*			
	Chowdhury et al. [211]	*N* = 150 AD and vascular neurocognitive disorder patients (>50 years). EGb 761: extract of *Ginkgo biloba* leaves: 240 mg/day. 18 weeks.	Significant improvements compared to baseline were found in constructional praxis, memory, speed and executive functioning, and behavioral symptoms. In total, 22.0% of patients presented adverse drug reactions, with headache and diarrhea being the most frequent events.
	García-Alberca et al. [212]	*N* = 133 MCI patients (64–84 years). EGb 761: 240 mg/day + acetylcholinesterase inhibitors: donepezil (5–10 mg daily), galantamine (16–24 mg daily), or rivastigmine patch (9.5–13.3 mg daily). 12 months.	Combined therapy with EGb 761 plus AChEI may provide added cognitive and functional benefits in patients with MCI.
	Herrschaft et al. [213]	*N* = 200 AD and vascular dementia patients (>60 years). EGb 761: 240 mg/day. 24 weeks.	Patients treated with EGb 761 improved the SKT total score. The NPI composite score improved in the EGb 761-treated group.
	Ihl et al. [214]	*N* = 202 AD and vascular dementia patients (>60 years). EGb 761: 240 mg/day. 24 weeks.	Patients treated with EGb 761 improved SKT and NPI total scores.
	Nikolova et al. [215]	*N* = 165 AD and vascular dementia patients (>60 years). EGb 761: 240 mg/day. 22 weeks.	EGb 761 improved impaired mitochondrial function, hippocampal neurogenesis, and neuroplasticity. It also inhibited the aggregation and toxicity of Aβ protein.
	Rapp et al. [216]	*N* = 189 AD patients (>80 years). EGb 761: 240 mg/day; donepezil (5–10 mg/day). 12 months.	Results suggest similar effects on cognitive symptoms from the use of EGb 761 in the treatment of dementia in AD, together with favorable safety compared to donepezil.
	Vellas et al. [217]	*N* = 1406 elderly participants who spontaneously reported memory complaints (>70 years). EGb 761: 240 mg/day. 5 years.	Long-term use of standardized Gb extract did not reduce the risk of progression to AD compared to placebo.
	Yancheva et al. [218]	*N* = 96 probable AD patients (>50 years). EGb 761: 240 mg/day, donepezil (5–10 mg/day), and both treatments. 22 weeks.	Changes from baseline to week 22 and response rates were similar for all three treatment groups with respect to all outcome measures (SKT, NPI, GBSS, HDRS, CDT, and Verbal Fluency Test).
Ginseng			
	Cheng et al. [219]	Effects of asiatic acid treatment on PC12 cells with Aβ25-35-induced injury.	Asiatic acid significantly increased the viability of differentiated PC12 cells but attenuated the mitochondria-mediated apoptosis dose-dependently when challenged with Aβ25-35. Asiatic acid protected differentiated PC12 cells from Aβ25-35-induced apoptosis and tau protein hyperphosphorylation, which might be partially mediated by the activation of the PI3K/Akt/GSK-3β signaling pathway.
	Guo et al. [220]	Effects of licochalcone A (LicA) treatment on SH-SY5Y cells with Aβ25-35-induced injury.	LicA improved cell viability and decreased lactate dehydrogenase leakage remarkably in Aβ25-35-induced injury in SH-SY5Y cells. After treatment with LicA, ROS, glutathione, and superoxide dismutase levels in cells all were significantly decreased, which indicated that LicA has an antioxidative effect on Aβ25-35-induced oxidative injury. LicA could also significantly reduce Aβ25-35-induced autophagy in SH-SY5Y cells. In the cells injured by Aβ25-35, LicA prevented the transformation from light chain protein 3-I to light chain protein 3-II and reduced the levels of proteins GRP78, GRP94, CHOP, and Bax, but increased the levels of antiapoptotic protein and phosphorylation of PI3K, Akt, and mTOR.
	Hu et al. [221]	Effects of WEG and ginsenoside Rb1 on SH-SY5Y cells treated with MPP.	WEG exhibited significant protective effects against MPP(+)-induced cytotoxicity in SH-SY5Y cells, possibly through the suppression of ROS generation and the inhibition of the mitochondria-dependent apoptotic pathway.
	Hwang et al. [222]	In vitro study utilizing SH-SY5Y cells treated with the ginsenoside Rb1.	Forty proteins were significantly changed in response to Rb1 treatment in β-amyloid-treated neuronal cells. Analysis of the interactions of altered proteins revealed that actin cytoskeleton proteins might be linked to the regulatory mechanisms of Rb1. The CAP1, CAPZB, TOMM40, and DSTN proteins showed potential as molecular target proteins for the functional contribution of Rb1 in AD.
	Liu et al. [223]	In vitro study utilizing SH-SY5Y cells treated with the ginsenoside Rd.	Rd could exert a beneficial effect in an experimental PD model in vitro, in which SH-SY5Y cells were injured by 1-methyl-4-phenylpyridinium (MPP+), an active metabolic product of the classical Parkinsonian toxin MPTP. Rd at 1 and 10 μM could significantly attenuate MPP+-induced cell death. This protective effect may be ascribed to its ability to reduce intracellular ROS levels, enhance antioxidant enzymatic activities, preserve the activity of respiratory complex I, stabilize the mitochondrial membrane potential, and increase intracellular ATP levels. Furthermore, the PI3K/Akt survival-signaling pathway was also involved in the protective effect of Rd.

AChEI: acetylcholinesterase inhibitors; AD: Alzheimer’s disease; ADAS-cog: Alzheimer’s Disease Assessment Scale—Cognitive Subscale; BADLS: Bristol’s Activities of Daily Living Scale; BASDEC: Brief Assessment Schedule Depression Cards; CDR: Clinical Dementia Rating Scale; CDT: Clock Drawing Test; CGI: Clinical Global Impression Scale; CUR: curcumin; DHA: docosahexaenoic acid; EPA: eicosapentaenoic acid; GBSS: Gottfries–Bråne–Steen Scale; HDRS: Hamilton Depression Rating Scale; HVLT-R: Hopkins Verbal Learning Test—Revised; LA: alpha lipoic acid; LXA_4_: lipoxin A_4_; MADRS: Montgomery–Asberg Depression Eating Scale; MCI: mild cognitive impairment; MPP+: 1-methyl-4-phenylpyridinium ion; MPTP: 1-methyl-4-phenyl-1,2,3,6-tetrahydropyridine; MMSE: Mini-Mental State Examination; NPI: Neuropsychiatric Inventory; PD: Parkinson’s disease; ROS: reactive oxygen species; RvD1: resolving D1; SKT: Syndrom–Kurztest Test; UPDRS: Unified Parkinson’s Disease Rating Scale.

## Abbreviations

The following abbreviations are used in this manuscript:

Aβ	Amyloid-β
AChE	Acetylcholinesterase
AD	Alzheimer’s disease
AX	Astaxanthin
BBB	Blood–brain barrier
BD	Bipolar disorder
BDNF	Brain-derived neurotrophic factor
CBD	Cannabidiol
CGA	Chlorogenic acid
CNS	Central nervous system
COX-2	Cyclooxygenase-2
CREB	cAMP response element binding protein
DHA	Docosahexaenoic acid
EFAs	Essential fatty acids
EPA	Eicosapentaenoic acid
ERK	Extracellular signal-regulated kinase
FMT	Fecal microbiota transplantation
Gb	*Ginkgo biloba*
GBA	Gut–brain axis
GM	Gut microbiome
GPR55	G-protein-coupled receptor 55
5-HT1A	Serotonin 1A receptor
iNOS	Inducible nitric oxide synthase
KRG	Korean red ginseng
LPS	Lipopolysaccharides
5-LOX	5-lipoxygenase
MAPK	Mitogen-activated protein kinase
MASLD	Metabolic dysfunction-associated steatotic liver disease
MDD	Major depressive disorder
NMDA	N-methyl-D-aspartate
NF-κB	Nuclear factor kappa-light-chain-enhancer of activated B cells
Nrf2/HO-1	Nuclear factor erythroid 2-related factor 2/heme oxygenase-1
omega-3 PUFAs	Omega-3 polyunsaturated fatty acids
omega-6 PUFAs	Omega-6 polyunsaturated fatty acids
PD	Parkinson’s disease
PKA	Protein kinase A
PKB	Protein kinase B
PPAR	Peroxisome proliferator-activated receptor
PPD	Protopanaxadiol
PPT	Protopanaxatriol
RCTs	Randomized clinical trials
ROS	Reactive oxygen species
SCFAs	Short-chain fatty acids
TDP-43	Trans-active response DNA-binding protein-43
THC	Δ9-tetrahydrocannabinol
TNF-α	Tumor necrosis factor-α
TQ	Thymoquinone
TRPC6	Transient receptor potential C6
